# The dark matter of the cancer genome: aberrations in regulatory elements, untranslated regions, splice sites, non‐coding RNA and synonymous mutations

**DOI:** 10.15252/emmm.201506055

**Published:** 2016-03-18

**Authors:** Sven Diederichs, Lorenz Bartsch, Julia C Berkmann, Karin Fröse, Jana Heitmann, Caroline Hoppe, Deetje Iggena, Danny Jazmati, Philipp Karschnia, Miriam Linsenmeier, Thomas Maulhardt, Lino Möhrmann, Johannes Morstein, Stella V Paffenholz, Paula Röpenack, Timo Rückert, Ludger Sandig, Maximilian Schell, Anna Steinmann, Gjendine Voss, Jacqueline Wasmuth, Maria E Weinberger, Ramona Wullenkord

**Affiliations:** ^1^ Division of Cancer ResearchDepartment of Thoracic SurgeryMedical CenterFaculty of MedicineUniversity of FreiburgFreiburgGermany; ^2^ Division of RNA Biology & Cancer (B150)German Cancer Research Center (DKFZ)HeidelbergGermany; ^3^ German Cancer Consortium (DKTK)FreiburgGermany; ^4^ German Academic Scholarship Foundation ‐ Studienstiftung des deutschen VolkesBonnGermany

**Keywords:** alternative polyadenylation, enhancer, mutation, non‐coding RNA, synonymous mutation, Cancer, Chromatin, Epigenetics, Genomics & Functional Genomics

## Abstract

Cancer is a disease of the genome caused by oncogene activation and tumor suppressor gene inhibition. Deep sequencing studies including large consortia such as TCGA and ICGC identified numerous tumor‐specific mutations not only in protein‐coding sequences but also in non‐coding sequences. Although 98% of the genome is not translated into proteins, most studies have neglected the information hidden in this “dark matter” of the genome. Malignancy‐driving mutations can occur in all genetic elements outside the coding region, namely in enhancer, silencer, insulator, and promoter as well as in 5′‐UTR and 3′‐UTR. Intron or splice site mutations can alter the splicing pattern. Moreover, cancer genomes contain mutations within non‐coding RNA, such as microRNA, lncRNA, and lincRNA. A synonymous mutation changes the coding region in the DNA and RNA but not the protein sequence. Importantly, oncogenes such as *TERT* or *miR‐21* as well as tumor suppressor genes such as *TP53*/*p53*,*APC*,*BRCA1,* or *RB1* can be affected by these alterations. In summary, coding‐independent mutations can affect gene regulation from transcription, splicing, mRNA stability to translation, and hence, this largely neglected area needs functional studies to elucidate the mechanisms underlying tumorigenesis. This review will focus on the important role and novel mechanisms of these non‐coding or allegedly silent mutations in tumorigenesis.

**Glossary**
Acceptor splice site: Splice site at the end of an intron (3′ end).
AU‐rich elements (ARE): Conserved motif of adenine/uridine bases in the 3′‐untranslated region (UTR) of an mRNA controlling mRNA decay.
Branch point: Sequence within the intron needed during splicing for the creation of the lariat structure. The adenine of the branch point forms a phosphodiester bond with the 5′ end of the intron.
*Cis*‐acting element: A non‐coding sequence in a gene or transcript with regulatory effects on the same or a nearby gene (*in cis*).
Consensus splice site: Nucleotide sequences that serve as splice sites in the majority of premature gene transcription. These include the highly conserved dinucleotides GT (5′ end of intron) and AG (3′ end of intron).
Cryptic splice site: Inactive splice site which can be activated when the previous dominant splice site loses its function.
Donor splice site: Splice site at the beginning of an intron (5′ end).
Driver mutation: Mutation that confers a growth advantage for the tumor leading to malignant initiation, promotion, or progression.
Epigenetic events: Events of gene regulation without underlying alterations in the DNA sequence, for example, through DNA methylation or histone modification.
Enhancer: Transcription factor binding site located up to 1 Mbp up‐ or downstream of a particular gene with bidirectional effects. The binding of a transcription factor to an enhancer results in the upregulation of the transcription of the respective gene.
Exon skipping: Exons are sequences that are usually retained during the splicing process and are part of the mature transcript. Exon skipping denotes a form of alternative splicing in which an exon and its neighboring introns are spliced out, for example, due to mutations in or different strengths of splice sites.
Insulator: Genomic region that creates a boundary between an enhancer and neighboring genes. Enhancer‐blocking insulators limit the number of genes which an enhancer can influence through selective disruption of enhancer–promoter interaction.
Internal ribosome entry site (IRES): Alternate ribosomal binding site (RBS) in mRNA, downstream of the classic RBS at the 5′ cap.
Intron retention: Introns are sequences that are usually cut out during the splicing process and are not part of the mature transcript. Intron retention denotes a form of alternative splicing in which whole or parts of introns remain in the RNA, for example, due to mutations in or different strengths of splice sites.
Kozak consensus sequence: A nucleotide sequence motif in mRNA essential for ribosomal assembly and initiation of translation around the start codon.
Long non‐coding RNA (lncRNA): Long non‐coding RNAs are non‐coding transcripts with a length of > 200 nucleotides and lacking a significant coding potential. LncRNAs affect a variety of cellular functions: they regulate gene expression, influence the activity and localization of proteins or nucleic acids, or act as scaffolds for the formation of cellular substructures and protein complexes.
microRNA (miRNA): Short, non‐coding RNA (18–25 nt) that can repress gene expression at the post‐transcriptional level by binding to mRNAs.
NCI‐60 Panel: A panel of the US National Cancer Institute comprising 60 different, well‐characterized human cancer cell lines that is used to test natural and chemical products and serves as a tool in cancer research.
Passenger mutations: Mutation that does not promote the fitness of malign cells or even damage them.
PIWI‐interacting RNAs or piRNAs: A class of small non‐coding RNAs mainly involved in the silencing of transposable elements (TEs) in germ cells.
Polyadenylation: After cleavage of a pre‐mRNA at its 3′‐end to terminate the transcript, roughly 250 adenosines are attached to the mRNA sequence that form the poly(A) tail ensuring translational efficacy and increasing mRNA stability.
Promoter: Region of DNA located within the close upstream area of a gene that contains binding sites for specific transcription factors crucial for the initiation of transcription.
Seed region: Nucleotides 2–8 of a microRNA largely determining target recognition by usually perfect complementarity to the target mRNA.
Single nucleotide polymorphism (SNP): Single nucleotide variation in the genome that is found in at least 1% of the population.
Silent mutation: Base substitution anywhere in the genome without any effect on the amino acid sequence of coding genes, for example, mutations outside of genes or in regulatory elements or synonymous mutations.
Synonymous mutation: Base substitution in the coding sequence of a protein‐coding gene that does not modify the amino acid sequence of the gene product due to the redundancy of the genetic code.
*Trans*‐acting element: A factor, usually a protein or oligonucleotide, with regulatory effects on a gene distant from its transcriptional source (*in trans*).
Upstream open reading frame (uORF): Open reading frame in the 5′‐UTR with regulatory effects on the translation of the main ORF downstream on the same mRNA

## Introduction

Cancer remains one of the leading causes of death worldwide according to the World Cancer Report 2014 (Stewart & Wild, 2014). Already in 1902, Theodor Boveri speculated that cancer might be a disease of the genome (Boveri, 2008). Research of the last decades confirmed this hypothesis and deepened our understanding of the genomic landscape of cancer (Alexandrov *et al*, 2013; Weinstein *et al*, 2013). We now know that a broad spectrum of molecular events can drive tumorigenesis. Genetic events range from amplifications, deletions, insertions, translocations, loss of heterozygosity to missense, non‐sense, or frameshift point mutations (Stratton *et al*, 2009; Vogelstein *et al*, 2013). Both, activated oncogenes and inactivated tumor suppressor genes, can contribute to tumorigenesis and progression by conferring tumor‐specific properties, called the hallmarks of cancer (Hanahan & Weinberg, 2000). Also epigenetic events and infectious agents as the human papillomavirus can have a tumorigenic effect, but these are beyond the scope of this review (zur Hausen, 2009; Baylin & Jones, 2011).

Although substantial progress in understanding of the cancer driver events has led to the development of new targeted therapeutics (Druker *et al*, 2001a; Sordella *et al*, 2004), the last decade of research has revealed that the genomic landscape of cancer is substantially more complex than previously assumed. This has been largely driven by the introduction of high‐throughput next‐generation sequencing techniques, which unravel the extensive mutational heterogeneity of tumors (Leiserson *et al*, 2015). These techniques allow rapid sequencing of a large number of complete genomes so that an increasing amount of cancer genome data becomes available (Kandoth *et al*, 2013). International consortia are involved in the generation and structuring of the abundance of information (Lawrence *et al*, 2013). The Cancer Genome Atlas (TCGA) Research Network aims to analyze molecular tumor profiles, for example, by detecting patterns across different types of cancer (Weinstein *et al*, 2013). The International Cancer Genome Consortium (ICGC) coordinates large‐scale cancer genome studies at the genomic, epigenomic, and transcriptomic levels. Over 25,000 genomes from 50 different cancer types are being sequenced to improve therapy, prognosis, and discovery of new targets (ICGC, 2010). For example, the identification of new mechanisms contributing to medulloblastoma tumorigenesis led to novel targets for therapy (Jones *et al*, 2012). These large‐scale approaches show a large number of different mutations (Wood *et al*, 2007), but dissecting the role of individual mutations in this landscape as either driver or passenger mutations will pose the next challenge (Kandoth *et al*, 2013; Weinstein *et al*, 2013).

So far, cancer research has mostly focused on mutations that alter protein‐coding sequences. For example, the standard Catalogue Of Somatic Mutations In Cancer (COSMIC) only lists aberrations in the coding sequences of genes (Forbes *et al*, 2008). However, this coding fraction only represents less than 2% of the human genome (Weinhold *et al*, 2014). Indeed, the vast majority of the genomic sequence is either transcribed into non‐coding RNAs or comprised of regulatory elements (Alexander *et al*, 2010). Nevertheless, this part of the genome has been mostly neglected as irrelevant for decades despite early examples of functional relevance, for example, of the non‐coding RNAs MALAT1 (Ji *et al*, 2003; Gutschner *et al*, 2013) or H19 (Gabory *et al*, 2006) (a comprehensive list of all gene names used in the review is provided in Table EV1).

The huge amount of sequence data now available provides the chance to explore the role of this dark matter in cancer genomes. In this review, we give a comprehensive overview on genetic aberrations not altering coding information and highlight the mechanisms whereby they nevertheless affect tumorigenesis. These include synonymous mutations as well as mutations in regulatory elements, untranslated regions, splice sites, and non‐coding RNAs.

## Regulatory elements

Functional mutations in regulatory regions, such as promoters and enhancers, can either create or destruct transcription factor (TF) binding sites. Additionally, structural aberrations such as translocations, deletions, insertions, or duplications can alter the interaction between regulatory elements and the coding genes they control. For example, strong promoters or enhancers brought into proximity of MYC or PAX5 can activate these oncogenes (Busslinger *et al*, 1996; Gerbitz *et al*, 1999).

Mutations occurring in regulatory regions—depending on whether the binding site of an activating or repressing transcription factor is affected—can result in transcriptional up‐ or downregulation. If oncogenes or tumor suppressor genes are affected, mutations in regulatory elements may constitute causative events in tumorigenesis.

In 2013, a promoter mutation was discovered in the telomerase reverse transcriptase (*TERT*) gene in melanoma patients (Horn *et al*, 2013). *TERT* encodes the catalytic subunit of telomerase, an enzyme that preserves the chromosomal ends, which would otherwise be shortened in each cell division. Aberrant *TERT* expression results in a limitless proliferative potential, a hallmark of cancer (Hanahan & Weinberg, 2000). The somatic transitions C228T and C250T in the *TERT* promoter do not only occur in melanoma, but strikingly in numerous malignancies such as hepatocellular carcinoma (HCC) and are among the most frequent mutations in cancer (Vinagre *et al*, 2013; Totoki *et al*, 2014; Weinhold *et al*, 2014; Melton *et al*, 2015). These mutations create a novel binding site for the ETS transcription factor GABP in the *TERT* promoter leading to an increased transcriptional activity (Bell *et al*, 2015). Consequently, these mutations constitute an important step in tumorigenesis. In addition, a synergistic interaction of the *TERT* promoter mutations with the *BRAF* V600E mutation that induces the ETS transcription factor possesses clinical relevance (Xing *et al*, 2014). Moreover, the mutated *TERT* promoter is a candidate biomarker for recurrence detection of urothelial carcinoma and thus constitutes a novel diagnostic tool (Kinde *et al*, 2013).

Mutations in regulatory regions can also cause the downregulation of tumor suppressors. In melanoma, three recurrent C > T transitions within the promoter region of the tumor suppressor gene *SDHD* disrupt ETS binding sites decreasing its transcription rate. These somatic promoter mutations correlate with a shorter overall survival in melanoma patients (Weinhold *et al*, 2014).

Enhancer mutations can likewise increase transcriptional levels of oncogenes. In T‐cell acute lymphoblastic leukemia (T‐ALL), a somatic heterozygous insertion creates a binding site for the transcription factor MYB. Thereby, a large regulatory element, a so‐called “super‐enhancer”, is created leading to the overexpression of the oncogene *TAL1* (Mansour *et al*, 2014). Another recent example is the germline single nucleotide polymorphism (SNP) rs2168101 G > T in a super‐enhancer within the first intron of *LMO1*. The G allele of this SNP constitutes a transcription factor binding site in the super‐enhancer that drives the expression of the oncogene *LMO1* and predisposes to neuroblastoma (Oldridge *et al*, 2015). The term super‐enhancer describes a large enhancer with extraordinarily high transcription factor enrichment (Pott & Lieb, 2015). Such super‐enhancers may serve as tumor‐specific targets and promising results have emerged in multiple myeloma, where selective super‐enhancer inhibition caused loss of oncogene expression (Loven *et al*, 2013).

Vice versa, downregulating mutations exist in enhancers. For example, the enhancer of the B‐cell differentiation factor *PAX5* is disrupted by somatic mutations, impairing the maturation of B cells and promoting chronic lymphocytic leukemia (CLL) (Puente *et al*, 2015).

Lastly, deletions can also affect insulator regions. Deregulation of the *H19*/*IGF2* locus causes the Beckwith–Wiedemann syndrome, which can give rise to embryonic tumors such as Wilms' tumors. Germline microdeletions within the regulatory region of the *H19*/*IGF2* locus can affect the insulator function resulting in reversed enhancement of two genes (Sparago *et al*, 2004; Ideraabdullah *et al*, 2014).

In addition to the examples described above, other mutations and especially polymorphisms in regulatory regions of cancer genes are associated with tumorigenesis (Table 1).

**Table 1:** Alterations within regulatory DNA elements

Genetic event	Regulation	Affected gene	Gene function	Alteration	Reference
New binding site for activating TF	↑	*TERT (M)*	Catalytic subunit of telomerase	C228T, C250T (promoter)	Bell *et al* (2015); Heidenreich *et al* (2014); Horn *et al* (2013)
		*TAL1 (M)*	Oncogene, transcription factor	insertion (super‐enhancer)	Mansour *et al* (2014)
		*MCL1 (M)*	Apoptosis inhibitor	insertion (promoter)	Moshynska *et al* (2004); Tobin *et al* (2005)
		*CCND1 (P)*	Oncogene, regulation of cell cycle progression	multiple SNPs (enhancer)	Schodel *et al* (2012)
		*MMP1 (P)*	MMP	(−1,607) 1G/2G (promoter)	Liu *et al* (2012)
		*HGF (P)*	Cell proliferation, survival, migration, and morphogenesis	truncation deletion (promoter)	Ma *et al* (2009b)
		*LMO1 (P)*	Transcription factor	SNP in super‐enhancer	Oldridge *et al* (2015)
New binding site for repressing TF	↓	*BRM (P)*	Cancer susceptibility gene	insertion (−741, −1,321) (promoter)	Gao *et al* (2013); Liu *et al* (2011); Wong *et al* (2014)
Disrupted binding site for activating TF	↓	*SDHD (M)*	Tumor suppressor gene, subunit of succinate dehydrogenase complex	3 hotspots C > T (promoter)	Weinhold *et al* (2014)
		*WDR74 (M)*	Cell cycle control, apoptosis	52 hotspots C > T (promoter)	Weinhold *et al* (2014)
		*PAX5 (M)*	B cell differentiation factor	multiple mutations (enhancer)	Puente *et al* (2015)
		*CK‐19 (M)*	Tumor marker (NSCLC)	G (−99)C (promoter)	Fujita *et al* (2001)
		*MMP2 (P)*	MMP	C (−1,306)T (promoter)	Liu *et al* (2012)
Disrupted binding site for repressing TF	↑	*AMACR (P)*	Racemase in fat metabolism	germline deletion (promoter)	Zhang *et al* (2009b)
Disrupted insulator	↑/↓	*IGF2/H19 (M)*	Proliferation control	germline deletion (insulator)	Ideraabdullah *et al* (2014); Sparago *et al* (2004)
Unknown	↓	*PLEKHS1 (M)*	Largely unknown	23 hotspots C > T (promoter)	Weinhold *et al* (2014)
	↓	*CASP8 (P)*	Induction of apoptosis	−652 6N del (promoter)	de Martino *et al* (2013); Li *et al* (2010); Malik *et al* (2011); Wang *et al* (2009)
	↑	*NFKB1 (P)*	Transcription factor	insertion (promoter)	Fan *et al* (2011); Mohd Suzairi *et al* (2013); Tang *et al* (2010); Zhang *et al* (2009a)
	↓	*BRCA1 (P)*	Tumor suppressor, DNA repair gene	5‐kb deletion (promoter + 5′‐UTR)	Brown *et al* (2002)
	↓	*MMP3 (P)*	MMP	(−1,171) 5A/6A (promoter)	Liu *et al* (2012)
	↑	*MMP7 (P)*	MMP	A (−181)G (promoter)	Liu *et al* (2012)
	↑	*MMP9 (P)*	MMP	C (−1,562)T (promoter)	Liu *et al* (2012)

Mutations are marked with (M); polymorphisms are marked with (P).

TF, transcription factor; MMP, matrix metalloproteinase.

## 5′‐Untranslated regions (5′‐UTR)

**Figure 1:**
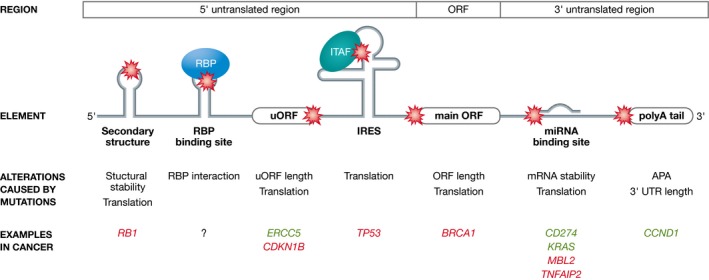
Schematic depiction of mutations within the 5′‐ and 3′‐UTR Mutations can alter the secondary structure of the 5′‐ or 3′‐UTR or occur in RNA binding protein (RBP) binding sites, upstream ORFs (uORF), internal ribosome entry sites (IRES; ITAF: IRES *trans*‐acting factor), start codons of open reading frames (ORF), microRNA binding sites, or polyadenylation signals (polyA). These alterations can affect translation efficiency, mRNA stability, ORF length, or RBP interaction as well as cause alternative cleavage and polyadenylation (APA). Prominent examples of genes involved in tumorigenesis (green: induced, red: decreased) that exhibit mutations (red star) in such elements are illustrated.

The untranslated regions (UTRs) flanking the coding region in mature messenger RNA (mRNA) regulate translation or mRNA stability through diverse mechanisms (Fig 1, Table 2). *Trans*‐acting RNA binding proteins (RBPs) and small RNAs can bind to either simple sequence elements or secondary and tertiary structures of the 5′‐UTR as well as the 3′‐UTR (reviewed in Di Liegro *et al*, 2014).

**Table 2:** Mutations and SNPs in 5′‐UTR elements associated with cancer

Gene	Variant	Regulatory element/Mechanism	Effect on protein	Cancer type	Reference
*CDKN1B*	4‐bp deletion C.‐456‐453del (g)	uORF	Decrease	MEN4	Occhi *et al* (2013)
*CDKN2A*	G‐34T (g)	Aberrant initiation codon	N/A	Melanoma	Liu *et al* (1999)
*C‐MYC*	C2756T (s)	IRES	Increase	Multiple myeloma	Chappell *et al* (2000)
*ERCC5*	A25G (SNP)	uORF	Increase	Pediatric ependymoma	Somers *et al* (2015)
*RAD51*	G135C (SNP)	Splice site/secondary structure	Decrease	Breast cancer	Antoniou *et al* (2007)
*RB1*	G17C, G18U (SNV, N/A)	Secondary structure	Decrease	Retinoblastoma	Kutchko *et al* (2015)
*TP53*	C119T (SNP)	IRES	Decrease	Melanoma	Khan *et al* (2013); Soto *et al* (2005)

Mutational status as indicated in (); s, somatic; g, germline; N/A, not available; SNP, single nucleotide polymorphism; SNV, single nucleotide variant.

*Cis*‐acting elements in the 5′‐UTR mediate translational regulation via the 5′‐cap or the secondary structure. Stable 5′‐UTR structures impede translation by reducing the accessibility for the translational machinery and ribosomal scanning. For example, mutations in *RB1* stabilize the 5′‐UTR secondary structures and are likely conducive to retinoblastoma (Kutchko *et al*, 2015). In addition, mutations in the Kozak consensus sequence can lead to leaky scanning and reduced translation initiation, for example, a somatic mutation in *BRCA1* in breast cancer (Signori *et al*, 2001; Wang *et al*, 2007).

Internal ribosome entry sites (IRES) allow cap‐independent translation—a mechanism crucial under cellular stress. A point mutation in the IRES *trans*‐acting factor binding domain of *TP53* reduces cap‐independent translation in steady‐state as well as under conditions of cellular stress (Khan *et al*, 2013) which may be linked to melanoma (Soto *et al*, 2005). Upstream open reading frames (uORFs) can reduce translation efficiency of the main open reading frame (ORF) or induce mRNA decay (reviewed in Barbosa *et al*, 2013). A germline mutation resulting in the deletion of a uORF stop codon in the *CDKN1B* gene shortens the intercistronic region and downregulates the translation of the main ORF in a case of multiple endocrine neoplasia syndrome type 4 (MEN4) (Occhi *et al*, 2013). In contrast, a common polymorphism in the 5′‐UTR of the *ERCC5* gene leads to the expression of a uORF (Somers *et al*, 2015). The translation of this uORF induces the expression of ERCC5 protein leading to resistance to platinum‐based chemotherapy and decreased survival in pediatric ependymoma (Somers *et al*, 2015). Alternatively, mutations within the 5′‐UTR can create aberrant initiation codons. A premature start codon by a germline mutation altering *CDKN2A* predisposes to melanoma (Liu *et al*, 1999). Other examples for frequent mutations in the 5′‐UTR still await functional characterization such as a somatic mutation in *BCL6* in non‐Hodgkin lymphoma (Migliazza *et al*, 1995).

## Synonymous mutations

**Figure 2:**
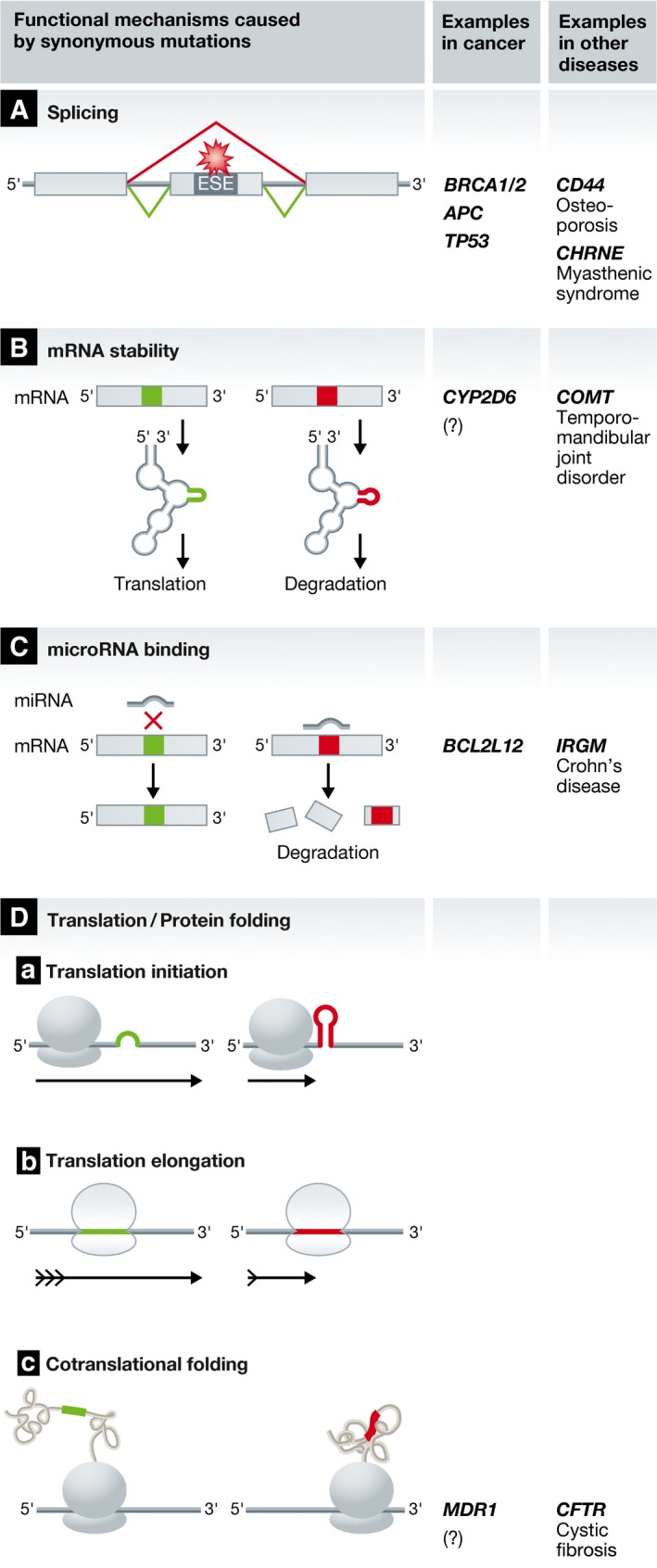
Functional mechanisms caused by synonymous mutations (A) Synonymous mutations can affect mRNA splicing. Of the possible splice events, an example of exon skipping by deletion of an exonic splicing enhancer (ESE) is shown (red). (B) Synonymous mutations can alter mRNA stability by modifying the secondary mRNA structure and lead to either translation (green) or mRNA degradation (red). (C) Protein abundance can be altered by either creating (red) or abrogating (green) a miRNA binding site in the mRNA. (D) Synonymous mutations can affect translation at different stages: (Da) translation initiation is favored by a less complex mRNA secondary structure (green) and hindered by a more stable secondary structure around the start codon (red). (Db) Codon choice and tRNA availability can either increase (green) or decrease (red) translational elongation speed. (Dc) The removal or creation of a ribosomal pause site can alter the protein conformation and structure. A ribosomal pause site in between two domains allows the translated domains to fold independently (green). The removal of a ribosomal pause site allows the cooperate folding of the two domains (red).

While silent mutations refer to all mutations not altering the amino acid sequence of a coding gene including mutations outside of genes or in regulatory elements or UTRs, synonymous mutations are a specific subset of silent mutations in which the mutation occurs in the coding region of a gene but does not alter the amino acid sequence due to the redundancy of the genetic code. In the past, synonymous mutations have been presumed to exert no functional effect. However, they are subject to natural selection in many species and are therefore likely to be functional (Drummond & Wilke, 2008; Supek *et al*, 2010). A few examples also indicate a role for synonymous mutations in diseases (Supek *et al*, 2014) (Fig 2, Table 3). In cancer, synonymous mutations are estimated to account for 20% of all point mutations, 6–8% of which are selected for and therefore may act as driver mutations (Supek *et al*, 2014). This selection occurs especially in oncogenes and is not reported for tumor suppressor genes, except for p53 (Supek *et al*, 2014).

**Table 3:** Examples for functions of synonymous mutations

Mechanism	Examples in cancer	Nucleotide change	Result	Examples in other disease
Splicing	*BRCA1/2* (Anczuków *et al*, 2008; Hansen *et al*, 2010; Raponi *et al*, 2011)	*BRCA1, 3719 G > T (g)* *BCRA2, 744 G > A (g)* *BCRA1, 231 G > T (g)*	Exon skipping *in vitro* Exon skipping Exon skipping	*CD44* (Vidal *et al*, 2009) (osteoporosis) *CHRNE* (Richard *et al*, 2007) (myasthenic syndrome)
	*APC* (Montera *et al*, 2001; Pećina‐Slaus *et al*, 2010)	*1869 G > T (g)* *5883 G > A (s)*	Exon skipping New splice site ?	
	*TP53* (Supek *et al*, 2014)	Multiple *(s)*	Multiple	
mRNA stability	*CYP2D6* (Toscano *et al*, 2006)	*2939 G > A (s)*	Predicted changes in mRNA structure may affect stability	*COMT* (Nackley *et al*, 2006) (temporo‐mandibular joint disorder)
microRNA binding	*BCL2L12* (Gartner *et al,* 2013)	*51 C > T (s)*	Loss of has‐miR‐671‐5p binding site	*IRGM* (Brest *et al*, 2011) (Crohn's disease)
Translation/Protein folding	*MDR1* (Kimchi‐Sarfaty *et al*, 2007)	*3435 C > T (s)*	Rare codon might lead to changes in cotranslational folding	*CFTR* (Bartoszewski *et al*, 2010; Lazrak *et al*, 2013) (cystic fibrosis)

The examples for the functions of synonymous mutations in cancer and other diseases are listed including the respective references. (g) Germline; (s) somatic.

Synonymous polymorphisms can correlate with the clinical outcome or therapy response and therefore serve as biomarkers; for example, they are associated with an increased risk of renal cell carcinoma recurrence (Schutz *et al*, 2013), with Gefitinib response in non‐small‐cell lung cancer (Ma *et al*, 2009a), or with Herceptin response in breast cancer (Griseri *et al*, 2011).

Synonymous substitutions can have functional consequences affecting various steps of protein biosynthesis resulting in changes in protein abundance and structure (reviewed in Hunt *et al*, 2014; Supek *et al*, 2014). Mechanisms include disruption or creation of splicing regulatory sites, alterations of mRNA stability, gain or loss of miRNA binding sites, and changes in translation efficiency. Although several functional mechanisms have been invoked in different diseases, only few are elucidated in cancer (Fig 2).

The most frequently reported mechanisms are related to dysfunctional splicing in tumor suppressors. Synonymous mutations can target exonic splicing regulatory sites, namely exonic splicing enhancers (ESE) and exonic splicing silencers (ESS). These motifs play a crucial role in identifying correct splice sites and when eliminated can lead to exon skipping, ectopic splice sites or activation of cryptic splice sites and a subsequent change in protein structure (Cartegni *et al*, 2002). Fifteen percent of synonymous mutations/point mutations have been estimated to cause human genetic diseases due to splicing defects (Krawczak *et al*, 1992). *BRCA2* synonymous mutations result in exon skipping and protein truncation (Anczuków *et al*, 2008; Raponi *et al*, 2011) and could be disease causing (Hansen *et al*, 2010). Exon skipping in APC is found in familial adenomatous polyposis (FAP) and colon cancer patients (Montera *et al*, 2001). Moreover, a new splice site is created in APC in lung cancer patients (Pećina‐Slaus *et al*, 2010). Synonymous mutations can also drive tumorigenesis by splice site inactivation in the *TP53* (*p53*) gene (Supek *et al*, 2014).

Synonymous mutations in proto‐oncogenes can also be functional. In melanoma cells, a synonymous substitution causes increased mRNA stability of the oncogene *BCL2L12*. This is due to the loss of the microRNA *miR‐671‐5p* target site in the coding sequence (Gartner *et al*, 2013). Also, a synonymous change in *CYP2D6* leads to decreased mRNA expression, resulting in an impaired drug oxidation phenotype affecting therapy response. Speculatively, this synonymous mutation could alter the secondary structure of the mRNA leading to its degradation (Toscano *et al*, 2006).

Synonymous mutations could also affect translational speed and thus change cotranslational protein folding (Yu *et al*, 2015). When a synonymous substitution results in a rare codon, transfer RNA (tRNA) availability can decrease the translational speed. This difference in translational speed can be associated with alternative protein conformation since—for example—a domain may have more time to fold before the next domain is translated (Yu *et al*, 2015). Domains can fold differently by either experiencing or not experiencing stabilization from neighboring domains (Purvis *et al*, 1987; Sauna & Kimchi‐Sarfaty, 2011). Vice versa, the removal of a ribosomal pause site by a synonymous mutation can lead to an alternative protein conformation, allowing cooperative folding of two domains (Tsai *et al*, 2008). A nucleotide substitution in the *MDR1* gene alters the substrate specificity of this ABC transporter (ATP‐binding cassette transporter) that is involved in multi‐drug resistance of cancer cells potentially by giving rise to an alternative protein conformation (Kimchi‐Sarfaty *et al*, 2007).

Lastly, mutations in overlapping open reading frames (ORFs) may be synonymous in one, but can result in a missense mutation in another ORF (FitzGerald *et al*, 1996). The same might hold true for transcription in the antisense direction.

In the past, synonymous mutations were assumed to be randomly distributed and used as controls for comparing mutation frequencies (Kimura, 1977). However, the described examples demonstrate that synonymous mutations can be relevant in cancer initiation, progression, and therapy response. Mutational studies of the past as well as public databases should be re‐investigated in order to determine a potential bias due to the inappropriate use of synonymous mutations as controls and to prevent the loss of valuable information hiding in synonymous mutations.

## Splice sites and introns

Splicing is a highly regulated process which adds a layer of complexity to protein biosynthesis in eukaryotic cells (Padgett *et al*, 1986). It can be disrupted or altered by mutations in *trans*‐acting splicing factors or *cis*‐acting sequences in exons and introns. Splicing mutations are increasingly recognized as important contributors to disease and are often linked to cancerogenesis (Wang & Cooper, 2007; Sterne‐Weiler & Sanford, 2014). Unlike mutations in splice factors and splicing mutations in exons, intronic mutations outside of the coding regions are often overlooked.

**Figure 3:**
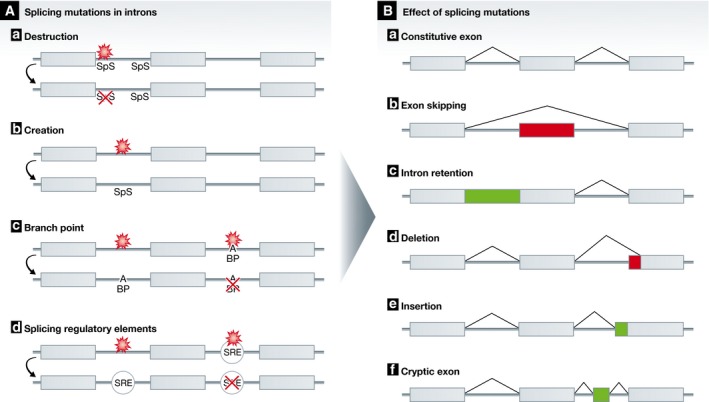
Sites and effects of intronic splicing mutations in cancer (A) Mutations in introns affecting splicing. a) Mutations in the intron can destroy a consensus splice donor or acceptor site (SpS) at the intron boundaries. b) Intronic mutations can create a novel splice donor or acceptor site (SpS). c) Mutations in introns can either create or destroy a branch point (BP). d) Mutations in splicing regulatory elements (SRE) can cause the formation or deletion of an intronic splicing silencer (ISS) or enhancer (ISE). (B) Products of splicing mutations. Depending on the presence of cryptic splice sites in the vicinity of the aforementioned mutations, the mutations can result in all product types of alternative splicing.

The majority of characterized intronic splicing mutations lead to the destruction or creation of consensus splice sites. Depending on the presence of cryptic splice sites, the outcome of these mutations can differ (Fig 3).

The destruction of splice sites is mostly caused by mutations in the highly conserved GT and AG dinucleotides at the 5′‐ or 3′‐intron‐ends, respectively. Splice site destruction can result in either deletion of the adjacent exon or retention of the adjacent intron. For example, a pathogenic donor splice site mutation in the tumor suppressor gene *MEN1* is responsible for the retention of intron 9 (Carrasco *et al*, 2004), while a mutation of a splice acceptor site in *CDKN2A* causes exon skipping in melanoma patients (Petronzelli *et al*, 2001). In *MLH1*, a T > A mutation in the polypyrimidine tract of a splice acceptor site provokes exon skipping classified as pathogenic in a hereditary non‐polyposis colon cancer (HNPCC) patient (Clarke *et al*, 2000). Although exon skipping is commonly caused by acceptor splice site mutations, donor splice site mutations can also account for exon skipping for instance in *WT1* (Schneider *et al,* 1993). Mutations in 5′ or 3′ splice sites are reported for the oncogene *MET* in lung cancer resulting in skipping of exon 14 (*MET*ex14). This leads to a functional protein lacking the binding site for the CBL E3 ligase. Hence, the oncoprotein partially escapes ubiquitination and degradation (Kong‐Beltran *et al*, 2006). Molecular profiling of lung adenocarcinoma reveals that 4% of tumors harbor *MET*ex14 (The Cancer Genome Atlas Research Network, 2014). Less frequently, destructions of splice sites are caused by mutations outside the GT and AG dinucleotides. In the presence of cryptic splice sites, these mutations can lead to deletion or partial intron retention in the processed mRNA transcript as the cryptic site becomes the novel used splice site. For example, a childhood adrenocortical tumor (ACT) harbors a splice acceptor site mutation in *TP53* which activates a downstream cryptic splice site leading to the deletion of the first ten nucleotides of exon 11 (Pinto *et al*, 2011). Vice versa, a G > A transition in the splice donor site in *TP53* results in the insertion of six amino acids in a patient with Li‐Fraumeni‐like syndrome (LFL syndrome) (Piao *et al*, 2013).

The creation of a novel splice site can render a consensus splice site cryptic. In a family with an attenuated retinoblastoma phenotype, a novel splice acceptor site in *RB1* mediates the insertion of four nucleotides in the mRNA (Sanchez‐Sanchez *et al*, 2005). In proximity to another cryptic splice site, one *de novo* splice site can create a cryptic exon. A *BRCA2* deep intronic mutation turns a cryptic splice site into a perfect consensus sequence (Anczukow *et al*, 2012). In several CLL cases, a point mutation in the 3′‐UTR of the *NOTCH1* gene creates a novel splice acceptor site which together with a cryptic splice site in the coding sequence leads to an additional splicing event and the deletion of 158 coding nucleotides including a PEST signal and hence leading to protein stabilization (Puente *et al*, 2015).

In addition to the destruction or creation of splice sites at the intron boundaries, mutations in the branch point can alter splicing. In *NF2*, a G > A transition creates a novel branch point, resulting in the usage of two cryptic splice sites which define a cryptic exon of 106 nucleotides (De Klein *et al*, 1998).

The last category of intronic mutations that affect splicing concerns splicing regulatory elements. In *ATM,* a cryptic exon is activated through a deep intronic four nucleotide deletion in an intron‐splicing processing element (ISPE) complementary to the U1 snRNP. The deletion disrupts this interaction which is sufficient to activate the cryptic exon (Pagani *et al*, 2002). Additionally, the role of synonymous mutations in exonic splicing enhancers or silencers has been discussed above.

Intronic splicing mutations classified as pathogenic are prevalent in many cancer types across a wide range of tumor suppressor genes (Sterne‐Weiler & Sanford, 2014). A more extensive overview on intronic splicing mutations in cancer can be found in Table EV2.

## 3′‐Untranslated regions (3′‐UTR)

As in the 5′‐UTR, elements in the 3′‐UTR can regulate translation and mRNA stability governing protein abundance (Fig 1).

The most prevalent class of regulatory elements in the 3′‐UTR are microRNA binding sites (miR‐BS). MicroRNAs (miRNAs) are small non‐coding RNAs regulating the majority of protein‐coding genes (Friedman *et al*, 2009; reviewed in Winter *et al*, 2009) by repressing translation, degrading mRNA by cleavage, or destabilizing it via deadenylation (Bartel, 2009; Fabian *et al*, 2010). Genetic aberrations in miR‐BS are frequent, but only functionally analyzed for individual examples (Table EV3).

Many cancer types show an enrichment of polymorphisms and mutations in miR‐BS. The most prominent example is a germline SNP in the 3′‐UTR of the oncogene *KRAS* known as LCS6 in the binding site for the *LET‐7* miRNA (Chin *et al*, 2008). This polymorphism elevates expression levels of *KRAS* and is associated with an increased risk of non‐small‐cell lung cancer (NSCLC). The LCS6 variant and mutations in the coding region of *KRAS* are mutually exclusive in the NCI‐60 panel (National Cancer Institute 60 human tumor cell line panel) pointing toward its potential relevance in tumorigenesis (Kundu *et al*, 2012). Frequent somatic mutations in *CD274* decrease binding of *miR‐570* in several cancer entities (Wang *et al*, 2012b). Conversely, mutations in the 3′‐UTR can also increase affinity to miRNAs or introduce new miR‐BS. For the germline SNP rs10082466 T > C in *MBL2*, the C‐allele enhances the affinity to *miR‐25a/b* and increases the risk for colorectal cancer (Zanetti *et al*, 2012). A somatic mutation of the *TNFAIP2* gene in an acute myeloid leukemia (AML) patient results in a Dicer‐dependent repression, suggesting the creation of a new miR‐BS for a yet unidentified miRNA (Ramsingh *et al*, 2010). A bioinformatical analysis predicts over 600 somatic mutations in 3′‐UTRs to interfere with miRNA binding alone (Ziebarth *et al*, 2012), and many other examples propose that this provides a general mechanism during tumorigenesis.

Polyadenylation signals demarcate the 3′‐end of a transcript leading to its cleavage and polyadenylation (polyA) (Moore, 2005). Usage of upstream polyA sites within the 3′‐UTR causes alternative cleavage and polyadenylation (UTR‐APA). UTR‐APA can promote mRNA stability by the loss of mRNA destabilizing sites and results in elevated protein levels, as detected in the (proto‐)oncogenes cyclin D1 (*CCND1*) or *IGF2BP1*/*IMP1* (Mayr & Bartel, 2009). A mutation that creates a premature polyadenylation signal in *CCND1* shortens its 3′‐UTR and increases the risk of mantle cell lymphoma (Wiestner *et al*, 2007). APA within the coding region (CR‐APA) leads to truncated proteins (Rehfeld *et al*, 2014) (Table 4).

**Table 4:** Variants in the 3′‐UTR affecting polyadenylation

Gene	Variant	Mechanism	Expression/effect on protein	Cancer type	Reference
*CCND1*	Several genomic deletions in 3′‐UTR (N/A)	Premature polyadenylation	Increase by enhanced stability of truncated mRNA (lacking AU‐rich elements, loss of miR‐BS)	Mantle cell lymphoma (oncogenic risk)	Wiestner *et al* (2007)
	Small aberration within 3′‐UTR (320 bp from stop codon: single base insertion (A at position 1344), small deletion (3 bp at position 1,344–46), duplication in repetitive element in 3′‐UTR (N/A)	Creation of APA signals			
*MSH6*	Duplication of 20 bp close to the polyadenylation site (g)	Decreased efficiency of polyadenylation	Decrease	Lynch syndrome	Decorsiere *et al* (2012)
*TP53*	*rs78378222 A/C* (g: SNP)	Change within polyadenylation signal	Decrease	Cutaneous basal cell carcinoma, prostate cancer, colorectal adenoma, glioma	Stacey *et al* (2011)
*PSMD8* *TM9SF3* *CD59* *ANKH* *CIAO1* *SRSF5* *MRSP16* *NDUFA6*	(N/A)	APA Differential usage of polyadenylation sites	Increase by enhanced stability of truncated mRNA due to miR‐BS loss	Small intestinal neuroendocrine tumor	Rehfeld *et al* (2014)

Mutational status as indicated in bold in brackets; s, somatic; g, germline; N/A, not available.

APA, alternative polyadenylation.

AU‐rich elements (ARE) in the 3′‐UTR mediate mRNA degradation; however, no specific mutations have been reported to date. In larger deletions, the loss of other regulatory sites is considered to be more relevant (Deshpande *et al*, 2009; Dixon *et al*, 2013).

Lastly, mutations in the 3′‐UTR may also cause aberrant splicing as described above for *NOTCH1* in CLL (Puente *et al*, 2015).

## Non‐coding RNAs

Non‐coding RNAs (ncRNAs) are a heterogeneous class of transcripts with low protein coding potential involved in diverse cellular processes.

MicroRNAs (miRNAs) are small ncRNAs of 18–25 nucleotides. Guided by the seed region, miRNAs bind to complementary sites in mRNAs repressing their translation and reducing mRNA stability. miRNAs influence numerous cellular processes including cell cycle regulation, differentiation, and apoptosis and can therefore act as tumor suppressors or oncogenes (Winter *et al*, 2009). Consequently, alterations in miRNA genes could have a major impact on tumorigenesis.

miRNA genes are often located in unstable genomic regions whose deletion is frequently involved in malignancies (Calin *et al*, 2004). Deletion of *miR‐15/‐16* at chromosome 13q14 stimulates tumor development due to reduced *BCL2* inhibition and dysregulation of cell cycle genes in CLL (Calin *et al*, 2002; Cimmino *et al*, 2005; Klein *et al*, 2010). However, since this genetically unstable genomic region contains more than this miRNA gene, the *miR‐15/‐16* cluster might not be affected selectively and adjacent genes can also be part of the same minimally deleted region, for example, the tumor suppressor gene *DLEU7*. The deletion of *DLEU7* results in a dysregulated NF‐κB pathway and inhibition of apoptosis synergistically with BCL2 (Palamarchuk *et al*, 2010). *DLEU2* also localizes to this fragile site and acts as a MYC‐dependent host gene of *miR‐15/16* (Lerner *et al*, 2009). The combination and interplay of these gene deletions might be crucial for tumorigenesis.

The *miR‐486* gene is located at a fragile genomic site at chromosome 8p11. Physiologically, *miR‐486* functions as a tumor suppressor and inhibits the anti‐apoptotic protein OLFM4. In up to 30% of gastric cancers, *miR‐486* is deleted, increasing cell proliferation and contributing to tumorigenesis (Oh *et al*, 2011).

In contrast to deletions of entire miRNA genes, point mutations can affect either the miRNA precursor and its processing or the mature miRNA sequence and its target recognition. Several SNPs have been described in miRNA precursors, and numerous association studies are reporting—sometimes conflicting—results on cancer susceptibility (Slaby *et al*, 2012 and references therein). The level of association differs greatly among cancer types, ethnic groups, sex, and lifestyle factors (Wang *et al*, 2012a). Since many polymorphisms do not have functional consequences, experimental verification is necessary for each individual variation (Diederichs & Haber, 2006). Most functional polymorphisms, however, influence the processing of the miRNA precursor and alter the level of the mature miRNA (Ryan *et al*, 2010).

In ALL, a somatic 13A > G mutation in the *miR‐128b* gene reduces its processing efficiency and thus lowers the level of mature *miR‐128b* (Kotani *et al*, 2010). Reduced *miR‐128b* is associated with resistance to the standard therapeutic agent dexamethasone (Kotani *et al*, 2009), demonstrating the clinical implications of mutations in miRNA genes.

Two polymorphisms in the *miR‐125a* gene are associated with breast cancer (Li *et al*, 2009; Lehmann *et al*, 2013). The variants lead to decreased levels of mature *miR‐125a* and upregulation of its target *ERBB2* (Duan *et al*, 2007; Lehmann *et al*, 2013).

Mutations rarely occur in the seed region of the miRNAs (Saunders *et al*, 2007), altering their ability to bind to target mRNAs. Somatic seed mutations of *miR‐142‐3p* in diffuse large B‐cell lymphoma, AML, and CLL do not affect its expression level, but enable binding to the 3′‐UTR of the *ZEB2* mRNA and disrupt binding to its physiological targets *RAC1* and *ADCY9* mRNAs (Kwanhian *et al*, 2012; The Cancer Genome Atlas Research Network, 2013; Kminkova *et al*, 2014).

P‐element‐induced wimpy testis (PIWI)‐interacting RNAs (piRNAs) are a class of small non‐coding RNA molecules that have originally been identified in *Drosophila* and are mainly involved in the silencing of transposable elements (TEs), especially in germ cells (Girard *et al*, 2006). A germline SNP (rs1326306) in piRNA 021285 was associated with an increased likelihood for breast cancer (Fu *et al*, 2015). This variant resulted in enhanced invasiveness when transfected into the breast cancer cell line MCF7, in part attributed to altered DNA methylation patterns of the *ARHGAP11A* gene leading to increased expression levels. *ARHGAP11A* codes for a Rho GTPase‐activating protein that enhances invasiveness in colon and breast cancer. This is the first example for a piRNA variant implicated in human cancer, but a growing body of literature dealing with piRNAs in this context makes it probable that many more are to be discovered. Recently, 273 of 20,831 known human piRNAs were found to be expressed in numerous somatic tissues in tissue‐specific patterns, suggesting a role in the control of cellular identity (Martinez *et al*, 2015). In the same study, 522 piRNAs were expressed in tumor tissues, largely distinguishing malignant from non‐malignant tissues in a cancer type‐specific pattern. Together with findings designating an involvement in post‐transcriptional regulation of gene expression to piRNAs (reviewed in Watanabe & Lin, 2014), this underlines a functional role of piRNAs beyond the control of TEs and stresses their potential contribution to tumorigenesis.

Long non‐coding RNAs (lncRNAs) are involved in a variety of cellular functions, although the underlying mechanisms or disease‐causing events are not yet revealed in most cases (Tsai *et al*, 2011). lncRNA expression and function are associated with many types of cancer (Gutschner & Diederichs, 2012), but only very few examples have been studied for genetic alterations.

*HOTAIR* is a well‐characterized lncRNA which is part of the *HOXC* locus and regulates *HOXD* genes *in trans* (Rinn *et al*, 2007). *HOTAIR* is overexpressed in hepatocellular carcinoma and breast cancer where it is associated with metastasis and shortened life expectancy (Gupta *et al*, 2010; Yang *et al*, 2011). The SNP rs7958904 (C > G) in exon 6 alters the secondary structure of *HOTAIR* and decreases cellular growth. In consequence, the risk for heterozygous carriers to develop colorectal carcinomas might be reduced (Xue *et al*, 2015).

Outside of oncology, mutations in the lncRNA *RMRP* in patients with cartilage–hair hypoplasia changed its chromatin binding properties. This lncRNA together with its associated RNA helicase DDX5 was important for the transactivational activity of the transcription factor RORγt likely causing the disease and hence providing a new therapeutic option (Huang *et al*, 2015).

Regarding other non‐coding RNA species, no cancer‐associated mutations have been studied in detail in ribosomal RNA (rRNA), small nuclear RNA (snRNA), transfer RNA (tRNA), or circular RNA (circRNA). A deletion in the small nucleolar RNA (snoRNA) *U50* gene is frequently present in prostate and breast cancer (Dong *et al*, 2008, 2009), demonstrating that also other ncRNA entities than miRNAs and lncRNAs might be mutated in cancer.

## Conclusions & outlook

Clearly, in addition to the protein‐coding genes, the major non‐coding fraction of the genome can be affected by tumor‐promoting mutations. Their number and functional effects have been underestimated in the past (Weinhold *et al*, 2014). High‐throughput sequencing techniques that allow for rapid sequencing of a vast amount of cancer genomes is now allowing rapid advances in this field (Stratton *et al*, 2009). Although international consortia attempt to structure the vast quantity of information, in‐depth analyses of sequencing data outside of coding sequences are still lacking. Advanced *in silico* methods need to be developed to cope with the huge amount of sequencing results. Most published studies dealing with non‐coding alterations in cancer are merely associative and generally focus on germline polymorphisms instead of somatic mutations (Table 1). Even though the molecular mechanisms of many of these alterations are unknown, the existing examples provide sufficient evidence for their importance in cancer. Further investigations to identify the full number of mutations and to delineate their functional impact are required. In studies published so far, there is a strong bias toward mechanisms which are simple to analyze such as splicing and miRNA binding. For intronic mutations affecting splicing, advanced *in silico* techniques with refined parameters based on functional data allow for reliable predictions of pathogenic events (Xiong *et al*, 2015).

In contrast, underlying mechanisms of other elements affecting translation efficiency or mRNA stability, for example, synonymous mutations, UTR folding into stable structures, or RBP binding sites, have been mostly neglected. Although numerous lncRNAs are dysregulated in several cancer entities, much less is known about their pathological or physiological effects and genetic aberrations (Prensner & Chinnaiyan, 2011). Hence, large efforts are needed to comprehensively elucidate the function of these players in tumorigenesis.

In recent years, the emergence of targeted therapies has revolutionized the treatment of cancer. Imatinib, the prime example of targeted therapy, shows that a detailed understanding of the genetic changes in a specific tumor entity can strikingly increase the survival of patients (Druker *et al*, 2001b). However, breakthroughs in targeted therapy are still limited to a few examples and acquired resistance is a major challenge. The non‐coding genome might unravel novel mechanisms underlying tumorigenesis and provide new tumor‐specific targets. For example, *in vitro* and animal studies applying antisense oligonucleotide therapy to correct aberrant splicing show promising results (Anczukow *et al*, 2012; Staropoli *et al*, 2015). Furthermore, regulatory elements such as super‐enhancers constitute potential therapeutic targets as they influence critical oncogenic drivers (Loven *et al*, 2013). Additionally, novel therapeutic approaches aim to replace or inhibit deregulated non‐coding RNAs in tumor cells, especially targeting miRNAs as potent regulators of mRNA translation and stability (reviewed in Kasinski & Slack, 2011; Rothschild, 2014). These might also be used to target mutations in miRNA genes that affect the levels of mature miRNAs or that impact its function. Repressed tumor‐suppressive miRNAs can be replaced or expression or binding of an oncogenic miRNA to a target mRNA can be inhibited by, for example, so‐called antagomiRs or by introducing miRNA masks complementary to the specific miR‐BS (Garzon *et al*, 2010). Future challenges of miRNA therapy include miRNA stability, tissue‐specific delivery systems, and potential off‐target effects (Rothschild, 2014).

Mutational loss of a miR‐BS can stabilize oncogenic mRNAs leading to increased susceptibility to cancer. In colorectal cancer (CRC), a SNP at position 8473 (T8473C; rs5275) of the cyclooxygenase‐2 (*COX2*) gene represses binding of miR‐542‐3p (Moore *et al*, 2012). The treatment‐of‐choice for SNP rs5275 carriers are (selective) COX‐2 inhibitors to significantly reduce the risk or boost tumor regression of COX‐2‐dependent CRC (Wang & Dubois, 2010; Dixon *et al*, 2013). However, the clinical impact of the respective SNP is a matter of debate (Cox *et al*, 2004; Gong *et al*, 2009). Hence, a refined investigation of the patient's mutational status beyond classical exonic (driver) mutations can significantly improve clinical outcome.

Lastly, long non‐coding RNAs could be exploited therapeutically, as well (Sanchez & Huarte, 2013), for example, suppression of the lncRNA MALAT1 in lung cancer metastasis (Gutschner *et al*, 2013).

Next to therapeutic targets, novel biomarkers may be also hidden in the “dark matter” of the genome with potential impacts on cancer diagnosis, prognosis, and response prediction. Numerous differential expression patterns of miRNAs and lncRNAs have been published to date, but also differential or cancer‐specific piRNA expression is associated with clinical parameters such as recurrence free survival and TNM stage in gastric, breast, colon, and kidney cancers (reviewed in Ng *et al*, 2016). In contrast, genetic alterations in these genes have been much less studied as potential biomarkers despite their obvious advantage of increased stability compared to expression alterations. Thus, comprehensive research focusing on both relevance and mechanisms of the identified mutations as well as detection of new non‐coding alterations in cancer will be of utmost importance in the coming years.

## Conflict of interest

The authors declare that they have no conflict of interest. S.D. is a co‐owner of the siTOOLs Biotech GmbH, Martinsried, Germany, which is unrelated to the topic covered in this review article.

**Pending issues**

Comprehensive discovery, quantification, and cataloging of tumor‐associated aberrations beyond mutations altering the coding region of genes in cancer.

In‐depth functional characterization of aberrations in regulatory elements, untranslated regions, splice sites, non‐coding RNA.

Mechanistic understanding of the contribution of synonymous mutations in cancer genes.

## Supporting information



Table EV1

Table EV2

Table EV3
