# Supplementary material for: The dark matter of the cancer genome: aberrations in regulatory elements, untranslated regions, splice sites, non‐coding RNA and synonymous mutations
Source: EMBO Mol Med. 2016 Mar 18;8(5):442–57. doi: 10.15252/emmm.201506055 (PMC5126213; doi:10.15252/emmm.201506055)
Supplement: Supplementary file 1 — Table EV1 [file EMMM-8-442-s001.docx]

**Table EV1: Gene List**

| **Gene Abbreviation** | **Full Gene Name** |
| --- | --- |
| *ADCY9* | Adenylate Cyclase 9 |
| *APC* | Adenomatous Polyposis Coli |
| *ATM* | Ataxia Telangiectasia Mutated |
| *BCL2* | B Cell Lymphoma 2 |
| *BCL2L12* | BCL2-like 12 |
| *BCL6* | B Cell Lymphoma 6 |
| *BRCA1* | Breast Cancer Associated Gene 1 |
| *BRCA2* | Breast Cancer Associated Gene 2 |
| *CCND1* | Cyclin D1 |
| *CD274* | Cluster of Differentation 274 |
| *CDKN1B* | Cyclin-Dependent Kinase Inhibitor 1B |
| *CDKN2A* | Cyclin-Dependent Kinase Inhibitor 2A |
| *COX2, PTGS2* | CycloOxygenase 2, Prostaglandin synthase 2 |
| *CYP2D6* | Cytochrome P450 2D6 |
| *DLEU2* | Deleted in Lymphocytic Leukemia 2 |
| *DLEU7* | Deleted in Lymphocytic Leukemia 7 |
| *DDX5* | Dead-box protein 5 (RNA helicase) |
| *ERBB2, HER2* | Human Epidermal Growth Factor Receptor 2 |
| *ERCC5* | Excision Repair Cross-Complementation Group 5 |
| *ETS* | E-Twenty-Six transformation specific (transcription factor) |
| *EXT1* | Exostosin-1 |
| *EXT2* | Exostosin-2 |
| *GABP* | GA Binding Protein (transcription factor) |
| *H19* | H19 imprinted ncRNA |
| *HOTAIR* | Homeobox Transcript Antisense Intergenic RNA |
| *HOXC* | Homeobox C |
| *HOXD* | Homeobox D |
| *IGF2* | Insulin-like Growth Factor 2 |
| *IGF2BP1, IMP-1* | Insulin-like Growth Factor 2 mRNA Binding Protein 1 |
| *KRAS* | Kirsten Rat Sarcoma viral oncogene homolog |
| *LET-7* | Lethal-7 |
| *LMO1* | LIM domain Only 1 |
| *MALAT1* | Metastasis Associated Lung Adenocarcinoma Transcript 1 |
| *MBL2* | Mannose-Binding Lectin 2 |
| *MDR1* | Multi-Drug Resistance gene 1 |
| *MEN1* | Multiple Endocrine Neoplasia Type 1 |
| *MET* | c-MET protooncogene |
| *MLH1* | MutL Homolog 1, DNA mismatch repair gene |
| *MYB* | Myeloblastosis (transcription factor) |
| *MYC* | Myelocytomatosis oncogene |
| *NF-κB, NFKB1* | Nuclear Factor kappa-light-chain-enhancer of activated B cells |
| *NF2* | Merlin or Neurofibromin 2 tumor suppressor |
| *NOTCH1* | Notch 1 |
| *OLFM4* | Olfactomedin4 |
| *PAX 5* | Paired Box 5 |
| *RAC1* | Ras-related C3 botulinum toxin substrate 1 |
| *RB1* | Retinoblastoma 1 |
| *RMRP* | RNA component of mitochondria RNA processing endoribonuclease |
| *RORɤt* | RAR-related Orphan Receptor (transcription factor) |
| *SDHD* | Succinate Dehydrogenase complex subunit D |
| *TAL1* | T cell Acute Lymphocytic Leukemia 1 |
| *TERT* | Telomerase Reverse Transcriptase |
| *TP53* | Tumor Protein p53 |
| *ZEB2* | Zinc Finger E-Box Binding Homeobox 2 |
