# Supplementary material for: The dark matter of the cancer genome: aberrations in regulatory elements, untranslated regions, splice sites, non‐coding RNA and synonymous mutations
Source: EMBO Mol Med. 2016 Mar 18;8(5):442–57. doi: 10.15252/emmm.201506055 (PMC5126213; doi:10.15252/emmm.201506055)
Supplement: Supplementary file 2 — Table EV2 [file EMMM-8-442-s002.docx]

**Table EV2: Intronic mutations affecting splicing in cancer**

| **Gene** | **Locus** | **Affected Site** | **Effect on Splicing** | **GermlineSomatic** | **Reference** |
| --- | --- | --- | --- | --- | --- |
| ***ATM*** | IVS10-6 T>G | n/a | exon skipping  (exon 11) | germline | (Broeks et al, 2003) |
|  | IVS28-159A>G | cryptic splice donor site | partial intron retention | germline | (Coutinho et al, 2005) |
|  | IVS20+12ins4 | U1 snRNP binding site | inclusion of a cryptic exon | germline | (Pagani et al, 2002) |
| ***BRCA1*** | c.4987-3C>G | n/a | exon skipping  (exon 17) | germline | (Brandao et al, 2012) |
|  | IVS5+3A>G | n/a | partial deletion of exon 5 | germline | (Claes et al, 1999) |
|  | IVS5+3A>G | n/a | n/a | germline | (De Leeneer et al, 2012) |
|  | (i)IVS5+1 G>T, (ii) IVS5+3 A>G, (iii)IVS21-6T>G | (i),(ii) splice donor site,  (iii) splice acceptor site | (i),(ii) partial exon skipping (exon 5), (iii) exon skipping (exon 21) | germline | (Meindl, 2002) |
|  | IVS8+2T>C | splice donor site | exon skipping  (exon 8) | germline | (Pyne et al, 1999) |
|  | IVS16+6T | cryptic splice donor site | partial intron retention | germline | (Scholl et al, 1999) |
|  | exon 12 4216-2nt A>G | splice acceptor site | retention of a single intronic base | germline | (Hartikainen et al, 2000) |
| ***BRCA2*** | c.8754+1 G>A | splice donor site | activation of a crytic exon | germline | (Hansen et al, 2008) |
|  | IVS6+2T>A | splice donor site | loss of exon 6 or loss of exons 6 and 5 or loss of exons 4 to 7 | germline | (Meindl, 2002) |
|  | IVS7+2 T>G | splice donor site | exon skipping  (exon 7) | germline | (Pyne et al, 2000) |
|  | c.7617+1G>A | splice donor site | exon skipping  (exon 15) | germline | (Thomassen et al, 2011) |
|  | IVS4-12del5 | polypyrimidine tract | exon skipping  (exon 5) | germline | (Zhang et al, 2009) |
|  | c.6937+594T>G | cryptic splice donor site | retention of a cryptic exon | germline | (Anczukow et al, 2012) |
| ***BUB1B*** | c.2386-11A>G | creation of a de novo splice site | partial intron retention | germline | (Rio Frio et al, 2010) |
| ***CDH1*** | c.833-2A>G | creation of a de novo splice acceptor site | partial intron retention | germline | (Kim et al, 2011) |
| ***CDKN2A*** | IVS1-1 G>C | splice acceptor site | exon skipping  (exon 2) | germline | (Petronzelli et al, 2001) |
|  | IVS2-105 A>G | splice donor site | creation of new splice donor site | germline | (Harland et al, 2001) |
|  | IVS1+37 G>C | cryptic splice site | inclusion of a cryptic exon | germline | (Balogh et al, 2012) |

**Table EV2: Intronic mutations affecting splicing in cancer (cont.)**

| **Gene** | **Locus** | **Affected Site** | **Effect on Splicing** | **GermlineSomatic** | **Reference** |
| --- | --- | --- | --- | --- | --- |
| ***CYLD*** | c.1139-148A>G | cryptic splice site | inclusion of new 65 nt out of frame exon | germline | (Kazakov et al, 2009) |
| ***ER*** | IVS5+/-(?)  A>G | cryptic splice donor site | inclusion of 69 nt | somatic | (Wang et al, 1997) |
| ***EXT1*** | c.1417+1  G>A | splice donor site | n/a | germline | (Philippe et al, 1997) |
| ***EXT2*** | IVS7+1G>T | splice donor site | n/a | germline | (Wolf et al, 1998; Yang et al, 2010) |
|  | c.1173+1G>T | splice donor site | exon skipping  (exon 7) | germline |  |
| ***INI1*** | c.500+1G>A | splice donor site | n/a | germline | (Ammerlaan et al, 2008) |
| ***MEN1*** | IVS9 + 1 G>A | splice donor site | Retention of intron 9 and partial exon skipping of exon 10 | germline | (Carrasco et al, 2004) |
|  | IVS7+2 T>G | splice donor site | n/a | germline | (Han et al, 2013) |
|  | 894-9 G>A | splice acceptor site | intron retention | germline | (Kishi et al, 1999) |
|  | IVS9-9 C>G | creation of a novel splice acceptor site | inclusion of eight additional nt | germline | (Lemos et al, 2007) |
|  | IVS4+/-(?)  G>A | splice acceptor site | n/a | germline | (Turner et al, 2002) |
| ***MET*** | IVS14+/-(?) | splice donor site | exon skipping  (exon 14) | somatic | (Kong-Beltran et al, 2006) |
| ***MLH1*** | IVS9 c.790+4A>T | splice donor site | exon skipping  (exon 9 and 10) | germline | (Bianchi et al, 2011) |
|  | IVS1-11T>A | splice acceptor site | exon skipping  (exon 2) | germline | (Clarke et al, 2000) |
|  | IVS2+1  del2 | splice donor site | exon skipping  (exon 2) | germline | (Gonda et al, 2002) |
|  | IVS9+2T>A | splice donor site | n/a | germline | (Han et al, 1995) |
|  | c.790+5G>T | splice donor site | n/a | germline | (Takahashi et al, 2012) |
|  | IVS6-2A>G | splice acceptor site | exon skipping  (exon 7) | germline | (Tanko et al, 2002) |
|  | c.589-2A>G | splice acceptor site | exon skipping  (exon 8) | germline | (Tomsic et al, 2012) |
| ***MSH2*** | c.942+3A>T | splice donor site | exon skipping  (exon 5) | germline | (Desai et al, 2000) |
|  | c.943+3A>T | splice donor site | exon skipping  (exon 5) | germline | (Froggatt et al, 1999) |
|  | c.[2635-3C>T;2635-5T>C] | splice acceptor site | n/a | germline | (Menendez et al, 2010) |
|  | IVS1-478 | splice donor site | Inclusion of a cryptic exon | germline | (Clendenning et al, 2011) |

**Table EV2: Intronic mutations affecting splicing in cancer (cont.)**

| **Gene** | **Locus** | **Affected Site** | **Effect on Splicing** | **GermlineSomatic** | **Reference** |
| --- | --- | --- | --- | --- | --- |
| ***MUTYH*** | IVS10+3A>C | splice donor site | exon skipping (exon 10) +/- intron retention (intron 11) | germline | (Pin et al, 2013) |
| ***NF1*** | (i) 47768A>G, (ii) IVS16+3del6,  (iii) IVS16-6del4, (iv) IVS19g-3C>G,  (v) IVS26-2A>T,  (vi) IVS27b-2A>T,  (vii) IVS39-12T>A | (i),(iii)-(vii) splice acceptor site,  (ii) splice donor site, | (i) insertion of 4nt, (ii) deletion of the last 233 nt within exon 16,  (iii) exon skipping (exon 17),  (iv) exon skipping (exon 20),  (v) insertion of 14 or 17 nt,  (vi) exon skipping (exon 28) or deletion of the first 293 nt of exon 28,  (vii) exon skipping (exon 40) or insertion of the last 10 nt of intron 39 | (i) germline, (ii) sporadic, (iii, iv) germline,  (v) sporadic,  (vi, vii) germline | (Ainsworth et al, 1994; Messiaen et al, 2000) |
|  | (i) IVS10b+ 1G>A,  (ii) IVS12a+1G>A | splice donor site | (i) exon skipping (exon 10b),  (ii) exon skipping (exon 11 and 12a) | sporadic | (Ars et al, 1999; Fang et al, 2001) |
| ***NF2*** | IVS15+10 ins200 | splice donor site | partial intron retention of about 200 nt | germline | (Ruttledge et al, 1996) |
|  | IVS6-18G>A | branch point creation | inclusion of a cryptic exon (exon 5a) | germline | (De Klein et al, 1998) |
| ***NOTCH1*** | 139390152T>C | cryptic splice acceptor site | deletion of the last 158 coding bases | sporadic | (Puente et al, 2015) |
| ***PMS2*** | IVS10-1 G>T  c989-1 G>T | splice acceptor site | (i) exon skipping (exon 10),  (ii) deletion of the first 27 nt of exon 10 | germline | (Sjursen et al, 2009) |
|  | c.2002A>G | splice acceptor site | 5nt deletion at the end of exon 11 | germline | (Li et al, 2015) |
| ***PTCH1*** | (i) c.1068-10T>A,  (ii) c.2561-2057A>G | cryptic splice acceptor site | (i) insertion of 8 nt, (ii) inclusion of a cryptic exon with 78 nt | germline | (Bholah et al, 2014) |
| ***PTEN*** | IVS7+7A>G | splice donor site | (i) exon skipping (exon 6),  (ii) deletion of the last 54 nt of exon 6 and deletion of the first 131 nt of exon 8 | germline | (Kurose et al, 2000) |

**Table EV2: Intronic mutations affecting splicing in cancer (cont.)**

| **Gene** | **Locus** | **Affected Site** | **Effect on Splicing** | **GermlineSomatic** | **Reference** |
| --- | --- | --- | --- | --- | --- |
| ***RAD51B*** | c.452+3A>G | splice donor site | exon skipping  (exon 5) | germline | (Golmard et al, 2013) |
| ***RAD51C*** | (i) c.706-2A > G  (ii) c.1026 + 5_1026 + 7del | splice donor site | (i) exon skipping (exon 5)  (ii) exon skipping (exon 8) | germline | (Golmard et al, 2013) |
| ***RB1*** | IVS23-1398A>G | cryptic splice donor site | partial intron retention | germline | (Dehainault et al, 2007) |
|  | c.2490-6T>A | cryptic splice acceptor site | insertion of 4 nt | sporadic | (Sanchez-Sanchez et al, 2005) |
|  | IVS8-10T>C | polypyrimidine tract of a splice acceptor site | exon skipping  (exon 9) | germline | (Lefevre et al, 2002) |
|  | IVS6+1G>T | splice donor site | exon skipping  (exon 6) | germline | (Klutz et al, 2002) |
| ***RecQ4*** | IVS8+31_41del | other intronic region | Retention of intron 8 | germline | (Balraj et al, 2002) |
|  | IVS12-1G>T | splice acceptor site | exon skipping  (exon 13) | germline | (Kitao et al, 1999) |
|  | IVS12-1G>A | splice acceptor site | 93 nt deletion | germline | (Lindor et al, 2000) |
| ***SUFU*** | c.1022 + 1G>A | splice donor site | exon skipping  (exon 8) | germline | (Pastorino et al, 2009) |
| ***TP53*** | IVS7-2A>C | splice acceptor site | different aberrant transcripts | somatic | (Bromidge et al, 2000) |
|  | IVS10-2 A > G | splice acceptor site | 10 nt deletion of exon 11 | germline | (Pinto et al, 2011) |
|  | IVS7+1G>T | splice donor site | retention of intron 7 | somatic | (Sameshima et al, 1990) |
|  | IVS3-1G>A | splice acceptor site | exon skipping and partial deletion of exon 4 | germline | (Varley et al, 1998) |
|  | c.671+1G >A | splice donor site | insertion of 6 nt | germline | (Piao et al, 2013) |
| ***TSC1*** | (i) IVS8+1G>A  (ii), IVS16+2T>C,(iii) IVS18-35T>C | (i) splice donor site, (ii) splice donor site, (iii) other intronic region | (i) exon skipping (exon 8), (ii) exon skipping (exon 16), (iii) 42 nt deletion | germline | (Mayer et al, 1999) |

**Table EV2: Intronic mutations affecting splicing in cancer (cont.)**

| **Gene** | **Locus** | **Affected Site** | **Effect on Splicing** | **GermlineSomatic** | **Reference** |
| --- | --- | --- | --- | --- | --- |
| ***TSC2*** | (i) IVS8+281C>T  (ii)IVS9-15G>A  (iii) IVS9-3C>G  (iv) IVS17-2A>G  (v) IVS24+1G>T  (vi) IVS37+1G>T  (vii) IVS38+16  del34bp  (viii) IVS38–18A>G | (i) cryptic splice donor site  (ii)-)(iv) splice acceptor site  (v),(vi) splice donor site  (vii),(viii) other intronic region | (i) insertion of 87 nt of intron 8  (ii),(iii) exon skipping (exon 10) and partial deletion of exon 10  (iv) 56 nt deletion of exon 18  (v) exon skipping (exon 24)  (vi) 29 nt deletion of exon 37  (vii) insertion of 75 nt of intron 38  (viii) retention of intron 38 |  | (Mayer et al, 1999) |
| ***WT1*** | IVS6+1G>C | splice donor site | exon skipping  (exon 6 or 5 and 6) | germline | (Schneider et al, 1993) |
|  | IVS7+2T>G | splice donor site | exon skipping  (exon 7) | germline (sporadic) | (Sakamoto et al, 2001) |
| ***XPA*** | IVS3-1G>C | splice acceptor site | 2 nt deletion of exon 4, sometimes exon skipping (exon 3) | germline | (Satokata et al, 1990)(Satokata et al, 1990)(Satokata et al, 1990) |
| ***XPC*** | IVS9+2T>G | splice donor site | exon skipping (exon 9) and in some cases insertion of 155 nt of intron 9 | germline | (Khan et al, 1998) |

**References Table EV2**

Ainsworth P, Rodenhiser D, Stuart A, Jung J (1994) Characterization of an intron 31 splice junction mutation in the neurofibromatosis type 1 (NF1) gene. *Hum Mol Genet* **3:** 1179-1181

Ammerlaan AC, Ararou A, Houben MP, Baas F, Tijssen CC, Teepen JL, Wesseling P, Hulsebos TJ (2008) Long-term survival and transmission of INI1-mutation via nonpenetrant males in a family with rhabdoid tumour predisposition syndrome. *Br J Cancer* **98:** 474-479

Anczukow O, Buisson M, Leone M, Coutanson C, Lasset C, Calender A, Sinilnikova OM, Mazoyer S (2012) BRCA2 deep intronic mutation causing activation of a cryptic exon: opening toward a new preventive therapeutic strategy. *Clin Cancer Res* **18:** 4903-4909

Ars E, Kruyer H, Gaona A, Serra E, Lazaro C, Estivill X (1999) Prenatal diagnosis of sporadic neurofibromatosis type 1 (NF1) by RNA and DNA analysis of a splicing mutation. *Prenat Diagn* **19:** 739-742

Balogh K, Szell M, Polyanka H, Pagani F, Bussani E, Kemeny L, Olah J (2012) Detection of a rare CDKN2A intronic mutation in a Hungarian melanoma-prone family and its role in splicing regulation. *Br J Dermatol* **167:** 131-133

Balraj P, Concannon P, Jamal R, Beghini A, Hoe TS, Khoo AS, Volpi L (2002) An unusual mutation in RECQ4 gene leading to Rothmund-Thomson syndrome. *Mutat Res* **508:** 99-105

Bholah Z, Smith MJ, Byers HJ, Miles EK, Evans DG, Newman WG (2014) Intronic splicing mutations in PTCH1 cause Gorlin syndrome. *Fam Cancer* **13:** 477-480

Bianchi F, Raponi M, Piva F, Viel A, Bearzi I, Galizia E, Bracci R, Belvederesi L, Loretelli C, Brugiati C, Corradini F, Baralle D, Cellerino R (2011) An intronic mutation in MLH1 associated with familial colon and breast cancer. *Fam Cancer* **10:** 27-35

Brandao RD, van Roozendaal KE, Tserpelis D, Caanen B, Gomez Garcia E, Blok MJ (2012) BRCA1 c.4987-3C>G is a pathogenic mutation. *Breast Cancer Res Treat* **131:** 723-725

Broeks A, Urbanus JH, de Knijff P, Devilee P, Nicke M, Klopper K, Dork T, Floore AN, van't Veer LJ (2003) IVS10-6T>G, an ancient ATM germline mutation linked with breast cancer. *Hum Mutat* **21:** 521-528

Bromidge T, Lowe C, Prentice A, Johnson S (2000) p53 intronic point mutation, aberrant splicing and telomeric associations in a case of B-chronic lymphocytic leukaemia. *Br J Haematol* **111:** 223-229

Carrasco CA, Gonzalez AA, Carvajal CA, Campusano C, Oestreicher E, Arteaga E, Wohllk N, Fardella CE (2004) Novel intronic mutation of MEN1 gene causing familial isolated primary hyperparathyroidism. *J Clin Endocrinol Metab* **89:** 4124-4129

Claes K, Machackova E, De Vos M, Poppe B, De Paepe A, Messiaen L (1999) Mutation analysis of the BRCA1 and BRCA2 genes in the Belgian patient population and identification of a Belgian founder mutation BRCA1 IVS5 + 3A > G. *Dis Markers* **15:** 69-73

Clarke LA, Veiga I, Isidro G, Jordan P, Ramos JS, Castedo S, Boavida MG (2000) Pathological exon skipping in an HNPCC proband with MLH1 splice acceptor site mutation. *Genes Chromosomes Cancer* **29:** 367-370

Clendenning M, Buchanan DD, Walsh MD, Nagler B, Rosty C, Thompson B, Spurdle AB, Hopper JL, Jenkins MA, Young JP (2011) Mutation deep within an intron of MSH2 causes Lynch syndrome. *Fam Cancer* **10:** 297-301

Coutinho G, Xie J, Du L, Brusco A, Krainer AR, Gatti RA (2005) Functional significance of a deep intronic mutation in the ATM gene and evidence for an alternative exon 28a. *Hum Mutat* **25:** 118-124

De Klein A, Riegman PH, Bijlsma EK, Heldoorn A, Muijtjens M, den Bakker MA, Avezaat CJ, Zwarthoff EC (1998) A G-->A transition creates a branch point sequence and activation of a cryptic exon, resulting in the hereditary disorder neurofibromatosis 2. *Hum Mol Genet* **7:** 393-398

De Leeneer K, Coene I, Crombez B, Simkens J, Van den Broecke R, Bols A, Stragier B, Vanhoutte I, De Paepe A, Poppe B, Claes K (2012) Prevalence of BRCA1/2 mutations in sporadic breast/ovarian cancer patients and identification of a novel de novo BRCA1 mutation in a patient diagnosed with late onset breast and ovarian cancer: implications for genetic testing. *Breast Cancer Res Treat* **132:** 87-95

Dehainault C, Michaux D, Pages-Berhouet S, Caux-Moncoutier V, Doz F, Desjardins L, Couturier J, Parent P, Stoppa-Lyonnet D, Gauthier-Villars M, Houdayer C (2007) A deep intronic mutation in the RB1 gene leads to intronic sequence exonisation. *Eur J Hum Genet* **15:** 473-477

Desai DC, Lockman JC, Chadwick RB, Gao X, Percesepe A, Evans DG, Miyaki M, Yuen ST, Radice P, Maher ER, Wright FA, de La Chapelle A (2000) Recurrent germline mutation in MSH2 arises frequently de novo. *J Med Genet* **37:** 646-652

Fang LJ, Simard MJ, Vidaud D, Assouline B, Lemieux B, Vidaud M, Chabot B, Thirion JP (2001) A novel mutation in the neurofibromatosis type 1 (NF1) gene promotes skipping of two exons by preventing exon definition. *J Mol Biol* **307:** 1261-1270

Froggatt NJ, Green J, Brassett C, Evans DG, Bishop DT, Kolodner R, Maher ER (1999) A common MSH2 mutation in English and North American HNPCC families: origin, phenotypic expression, and sex specific differences in colorectal cancer. *J Med Genet* **36:** 97-102

Golmard L, Caux-Moncoutier V, Davy G, Al Ageeli E, Poirot B, Tirapo C, Michaux D, Barbaroux C, d'Enghien CD, Nicolas A, Castera L, Sastre-Garau X, Stern MH, Houdayer C, Stoppa-Lyonnet D (2013) Germline mutation in the RAD51B gene confers predisposition to breast cancer. *BMC Cancer* **13:** 484

Gonda K, Nomizu T, Fukayama N, Sugano K, Takenosita S (2002) A novel germline mutation of hMLH1 in a patient with hereditary non-polyposis colorectal cancer. *Jpn J Clin Oncol* **32:** 215-218

Han B, Song ZY, Wu JJ, Liu W, Liu BL, Ye XP, Chen X, Pan CM, Xu HY, Li L, Zhu H, Lu YL, Wu WL, Chen MD, Song HD, Qiao J (2013) A novel intronic mutation and a missense mutation of MEN1 identified in two Chinese families with multiple endocrine neoplasia type 1. *J Endocrinol Invest* **36:** 162-167

Han HJ, Maruyama M, Baba S, Park JG, Nakamura Y (1995) Genomic structure of human mismatch repair gene, hMLH1, and its mutation analysis in patients with hereditary non-polyposis colorectal cancer (HNPCC). *Hum Mol Genet* **4:** 237-242

Hansen TV, Bisgaard ML, Jonson L, Albrechtsen A, Filtenborg-Barnkob B, Eiberg H, Ejlertsen B, Nielsen FC (2008) Novel de novo BRCA2 mutation in a patient with a family history of breast cancer. *BMC Med Genet* **9:** 58

Harland M, Mistry S, Bishop DT, Bishop JA (2001) A deep intronic mutation in CDKN2A is associated with disease in a subset of melanoma pedigrees. *Hum Mol Genet* **10:** 2679-2686

Hartikainen JM, Pirskanen MM, Arffman AH, Ristonmaa UK, Mannermaa AJ (2000) A Finnish BRCA1 exon 12 4216-2nt A to G splice acceptor site mutation causes aberrant splicing and frameshift, leading to protein truncation. *Hum Mutat* **15:** 120

Kazakov DV, Thoma-Uszynski S, Vanecek T, Kacerovska D, Grossmann P, Michal M (2009) A case of Brooke-Spiegler syndrome with a novel germline deep intronic mutation in the CYLD gene leading to intronic exonization, diverse somatic mutations, and unusual histology. *Am J Dermatopathol* **31:** 664-673

Khan SG, Levy HL, Legerski R, Quackenbush E, Reardon JT, Emmert S, Sancar A, Li L, Schneider TD, Cleaver JE, Kraemer KH (1998) Xeroderma pigmentosum group C splice mutation associated with autism and hypoglycinemia. *J Invest Dermatol* **111:** 791-796

Kim S, Ki CS, Kim KM, Lee MG, Kim S, Bae JM, Kim JW (2011) Novel mechanism of a CDH1 splicing mutation in a Korean patient with signet ring cell carcinoma. *BMB Rep* **44:** 725-729

Kishi M, Tsukada T, Shimizu S, Hosono K, Ohkubo T, Kosuge T, Sugano K, Kanbe M, Obara T, Yamaguchi K (1999) A novel splicing mutation (894-9 G --> A) of the MEN1 gene responsible for multiple endocrine neoplasia type 1. *Cancer Lett* **142:** 105-110

Kitao S, Shimamoto A, Goto M, Miller RW, Smithson WA, Lindor NM, Furuichi Y (1999) Mutations in RECQL4 cause a subset of cases of Rothmund-Thomson syndrome. *Nat Genet* **22:** 82-84

Klutz M, Brockmann D, Lohmann DR (2002) A parent-of-origin effect in two families with retinoblastoma is associated with a distinct splice mutation in the RB1 gene. *Am J Hum Genet* **71:** 174-179

Kong-Beltran M, Seshagiri S, Zha J, Zhu W, Bhawe K, Mendoza N, Holcomb T, Pujara K, Stinson J, Fu L, Severin C, Rangell L, Schwall R, Amler L, Wickramasinghe D, Yauch R (2006) Somatic mutations lead to an oncogenic deletion of met in lung cancer. *Cancer Res* **66:** 283-289

Kurose K, Zhou XP, Araki T, Eng C (2000) Biallelic inactivating mutations and an occult germline mutation of PTEN in primary cervical carcinomas. *Genes Chromosomes Cancer* **29:** 166-172

Lefevre SH, Chauveinc L, Stoppa-Lyonnet D, Michon J, Lumbroso L, Berthet P, Frappaz D, Dutrillaux B, Chevillard S, Malfoy B (2002) A T to C mutation in the polypyrimidine tract of the exon 9 splicing site of the RB1 gene responsible for low penetrance hereditary retinoblastoma. *J Med Genet* **39:** E21

Lemos MC, Harding B, Shalet SM, Thakker RV (2007) A novel MEN1 intronic mutation associated with multiple endocrine neoplasia type 1. *Clin Endocrinol* **66:** 709-713

Li L, Hamel N, Baker K, McGuffin MJ, Couillard M, Gologan A, Marcus VA, Chodirker B, Chudley A, Stefanovici C, Durandy A, Hegele RA, Feng BJ, Goldgar DE, Zhu J, De Rosa M, Gruber SB, Wimmer K, Young B, Chong G, Tischkowitz MD, Foulkes WD (2015) A homozygous PMS2 founder mutation with an attenuated constitutional mismatch repair deficiency phenotype. *J Med Genet* **52:** 348-352

Lindor NM, Furuichi Y, Kitao S, Shimamoto A, Arndt C, Jalal S (2000) Rothmund-Thomson syndrome due to RECQ4 helicase mutations: report and clinical and molecular comparisons with Bloom syndrome and Werner syndrome. *Am J Med Genet* **90:** 223-228

Mayer K, Ballhausen W, Rott HD (1999) Mutation screening of the entire coding regions of the TSC1 and the TSC2 gene with the protein truncation test (PTT) identifies frequent splicing defects. *Hum Mutat* **14:** 401-411

Meindl A (2002) Comprehensive analysis of 989 patients with breast or ovarian cancer provides BRCA1 and BRCA2 mutation profiles and frequencies for the German population. *Int J Cancer* **97:** 472-480

Menendez M, Castellvi-Bel S, Pineda M, de Cid R, Munoz J, Gonzalez S, Teule A, Balaguer F, Ramon y Cajal T, Rene JM, Blanco I, Castells A, Capella G (2010) Founder effect of a pathogenic MSH2 mutation identified in Spanish families with Lynch syndrome. *Clin Genet* **78:** 186-190

Messiaen LM, Callens T, Mortier G, Beysen D, Vandenbroucke I, Van Roy N, Speleman F, Paepe AD (2000) Exhaustive mutation analysis of the NF1 gene allows identification of 95% of mutations and reveals a high frequency of unusual splicing defects. *Hum Mutat* **15:** 541-555

Pagani F, Buratti E, Stuani C, Bendix R, Dork T, Baralle FE (2002) A new type of mutation causes a splicing defect in ATM. *Nat Genet* **30:** 426-429

Pastorino L, Ghiorzo P, Nasti S, Battistuzzi L, Cusano R, Marzocchi C, Garre ML, Clementi M, Scarra GB (2009) Identification of a SUFU germline mutation in a family with Gorlin syndrome. *Am J Med Genet* **149a:** 1539-1543

Petronzelli F, Sollima D, Coppola G, Martini-Neri ME, Neri G, Genuardi M (2001) CDKN2A germline splicing mutation affecting both p16(ink4) and p14(arf) RNA processing in a melanoma/neurofibroma kindred. *Genes Chromosomes Cancer* **31:** 398-401

Philippe C, Porter DE, Emerton ME, Wells DE, Simpson AH, Monaco AP (1997) Mutation screening of the EXT1 and EXT2 genes in patients with hereditary multiple exostoses. *Am J Hum Genet* **61:** 520-528

Piao J, Sakurai N, Iwamoto S, Nishioka J, Nakatani K, Komada Y, Mizutani S, Takagi M (2013) Functional studies of a novel germline p53 splicing mutation identified in a patient with Li-Fraumeni-like syndrome. *Mol Carcinog* **52:** 770-776

Pin E, Pastrello C, Tricarico R, Papi L, Quaia M, Fornasarig M, Carnevali I, Oliani C, Fornasin A, Agostini M, Maestro R, Barana D, Aretz S, Genuardi M, Viel A (2013) MUTYH c.933+3A>C, associated with a severely impaired gene expression, is the first Italian founder mutation in MUTYH-Associated Polyposis. *Int J Cancer* **132:** 1060-1069

Pinto EM, Ribeiro RC, Kletter GB, Lawrence JP, Jenkins JJ, Wang J, Shurtleff S, McGregor L, Kriwacki RW, Zambetti GP (2011) Inherited germline TP53 mutation encodes a protein with an aberrant C-terminal motif in a case of pediatric adrenocortical tumor. *Fam Cancer* **10:** 141-146

Puente XS, Bea S, Valdes-Mas R, Villamor N, Gutierrez-Abril J, Martin-Subero JI, Munar M, Rubio-Perez C, Jares P, Aymerich M, Baumann T, Beekman R, Belver L, Carrio A, Castellano G, Clot G, Colado E, Colomer D, Costa D, Delgado J, Enjuanes A, Estivill X, Ferrando AA, Gelpi JL, Gonzalez B, Gonzalez S, Gonzalez M, Gut M, Hernandez-Rivas JM, Lopez-Guerra M, Martin-Garcia D, Navarro A, Nicolas P, Orozco M, Payer AR, Pinyol M, Pisano DG, Puente DA, Queiros AC, Quesada V, Romeo-Casabona CM, Royo C, Royo R, Rozman M, Russinol N, Salaverria I, Stamatopoulos K, Stunnenberg HG, Tamborero D, Terol MJ, Valencia A, Lopez-Bigas N, Torrents D, Gut I, Lopez-Guillermo A, Lopez-Otin C, Campo E (2015) Non-coding recurrent mutations in chronic lymphocytic leukaemia. *Nature* **526:** 519-524

Pyne MT, Brothman AR, Ward B, Pruss D, Hendrickson BC, Scholl T (2000) The BRCA2 genetic variant IVS7 + 2T-->G is a mutation. *J Hum Genet* **45:** 351-357

Pyne MT, Pruss D, Ward BE, Scholl T (1999) A characterization of genetic variants in BRCA1 intron 8 identifies a mutation and a polymorphism. *Mutat Res* **406:** 101-107

Rio Frio T, Lavoie J, Hamel N, Geyer FC, Kushner YB, Novak DJ, Wark L, Capelli C, Reis-Filho JS, Mai S, Pastinen T, Tischkowitz MD, Marcus VA, Foulkes WD (2010) Homozygous BUB1B mutation and susceptibility to gastrointestinal neoplasia. *N Engl J Med* **363:** 2628-2637

Ruttledge MH, Andermann AA, Phelan CM, Claudio JO, Han FY, Chretien N, Rangaratnam S, MacCollin M, Short P, Parry D, Michels V, Riccardi VM, Weksberg R, Kitamura K, Bradburn JM, Hall BD, Propping P, Rouleau GA (1996) Type of mutation in the neurofibromatosis type 2 gene (NF2) frequently determines severity of disease. *Am J Hum Genet* **59:** 331-342

Sakamoto J, Takata A, Fukuzawa R, Kikuchi H, Sugiyama M, Kanamori Y, Hashizume K, Hata JI (2001) A novel WT1 gene mutation associated with wilms' tumor and congenital male genitourinary malformation. *Pediatr Res* **50:** 337-344

Sameshima Y, Akiyama T, Mori N, Mizoguchi H, Toyoshima K, Sugimura T, Terada M, Yokota J (1990) Point mutation of the p53 gene resulting in splicing inhibition in small cell lung carcinoma. *Biochem Biophys Res Commun* **173:** 697-703

Sanchez-Sanchez F, Kruetzfeldt M, Najera C, Mittnacht S (2005) A novel constitutional mutation affecting splicing of retinoblastoma tumor suppressor gene intron 23 causes partial loss of pRB activity. *Hum Mutat* **25:** 223

Satokata I, Tanaka K, Miura N, Miyamoto I, Satoh Y, Kondo S, Okada Y (1990) Characterization of a splicing mutation in group A xeroderma pigmentosum. *Proc Natl Acad Sci U S A* **87:** 9908-9912

Schneider S, Wildhardt G, Ludwig R, Royer-Pokora B (1993) Exon skipping due to a mutation in a donor splice site in the WT-1 gene is associated with Wilms' tumor and severe genital malformations. *Hum Genet* **91:** 599-604

Scholl T, Pyne MT, Russo D, Ward BE (1999) BRCA1 IVS16+6T-->C is a deleterious mutation that creates an aberrant transcript by activating a cryptic splice donor site. *Am J Med Genet* **85:** 113-116

Sjursen W, Bjornevoll I, Engebretsen LF, Fjelland K, Halvorsen T, Myrvold HE (2009) A homozygote splice site PMS2 mutation as cause of Turcot syndrome gives rise to two different abnormal transcripts. *Fam Cancer* **8:** 179-186

Takahashi M, Furukawa Y, Shimodaira H, Sakayori M, Moriya T, Moriya Y, Nakamura Y, Ishioka C (2012) Aberrant splicing caused by a MLH1 splice donor site mutation found in a young Japanese patient with Lynch syndrome. *Fam Cancer* **11:** 559-564

Tanko Q, Franklin B, Lynch H, Knezetic J (2002) A hMLH1 genomic mutation and associated novel mRNA defects in a hereditary non-polyposis colorectal cancer family. *Mutat Res* **503:** 37-42

Thomassen M, Pedersen IS, Vogel I, Hansen TV, Brasch-Andersen C, Brasen CL, Cruger D, Sunde L, Nielsen FC, Jensen UB, Bisgaard ML, Borg A, Gerdes AM, Kruse TA (2011) A BRCA2 mutation incorrectly mapped in the original BRCA2 reference sequence, is a common West Danish founder mutation disrupting mRNA splicing. *Breast Cancer Res Treat* **128:** 179-185

Tomsic J, Liyanarachchi S, Hampel H, Morak M, Thomas BC, Raymond VM, Chittenden A, Schackert HK, Gruber SB, Syngal S, Viel A, Holinski-Feder E, Thibodeau SN, de la Chapelle A (2012) An American founder mutation in MLH1. *Int J Cancer* **130:** 2088-2095

Turner JJ, Leotlela PD, Pannett AA, Forbes SA, Bassett JH, Harding B, Christie PT, Bowen-Jones D, Ellard S, Hattersley A, Jackson CE, Pope R, Quarrell OW, Trembath R, Thakker RV (2002) Frequent occurrence of an intron 4 mutation in multiple endocrine neoplasia type 1. *J Clin Endocrinol Metab* **87:** 2688-2693

Varley JM, McGown G, Thorncroft M, White GR, Tricker KJ, Kelsey AM, Birch JM, Evans DG (1998) A novel TP53 splicing mutation in a Li-Fraumeni syndrome family: a patient with Wilms' tumour is not a mutation carrier. *Br J Cancer* **78:** 1081-1083

Wang M, Dotzlaw H, Fuqua SA, Murphy LC (1997) A point mutation in the human estrogen receptor gene is associated with the expression of an abnormal estrogen receptor mRNA containing a 69 novel nucleotide insertion. *Breast Cancer Res Treat* **44:** 145-151

Wolf M, Hemminki A, Kivioja A, Sistonen P, Kaitila I, Ervasti H, Kinnunen J, Karaharju E, Knuutila S (1998) A novel splice site mutation of the EXT2 gene in a Finnish hereditary multiple exostoses family. Mutations in brief no. 197. Online. *Hum Mutat* **12:** 362

Yang L, Hui WS, Chan WC, Ng VC, Yam TH, Leung HC, Huang JD, Shum DK, Jie Q, Cheung KM, Cheah KS, Luo Z, Chan D (2010) A splice-site mutation leads to haploinsufficiency of EXT2 mRNA for a dominant trait in a large family with multiple osteochondromas. *J Orthop Res* **28:** 1522-1530

Zhang L, Bacares R, Boyar S, Hudis C, Nafa K, Offit K (2009) cDNA analysis demonstrates that the BRCA2 intronic variant IVS4-12del5 is a deleterious mutation. *Mutat Res* **663:** 84-89
