# Supplementary material for: The dark matter of the cancer genome: aberrations in regulatory elements, untranslated regions, splice sites, non‐coding RNA and synonymous mutations
Source: EMBO Mol Med. 2016 Mar 18;8(5):442–57. doi: 10.15252/emmm.201506055 (PMC5126213; doi:10.15252/emmm.201506055)
Supplement: Supplementary file 3 — Table EV3 [file EMMM-8-442-s003.docx]

**Table EV3: Mutations & SNPs in miRNA binding sites in 3’UTRs associated with cancer**

Only studies in which an effect on expression was confirmed by molecular biology methods were included. Endpoints are given as comparison between variant and control if not marked otherwise. OR=odds ratio; OS=overall survival; RR=relative risk; TTR=time to recurrence; TRD=tumor related death; HR=hazard ratio; †=replication studies in the same publication.

| **Gene** | **Variant** | **miRNA** | **Endpoint** | **Value of Endpoint** (95% CI) **/ Genotype** | **Expres-sion** | **Cancer type** | **Reference** |
| --- | --- | --- | --- | --- | --- | --- | --- |
| **BCL2L12** | C51T | miR-671–5p | Recurrence | 10 of 256 | Increase | Melanoma | (Gartner et al, 2013) |
| **BIRC5** | rs2239680  T>C | miR-335 | OR | 3.43 (2.04–5.77)/CC+CT  1.63 (1.17–2.26)†/CC+CT | Increase | Lung cancer | (Zu et al, 2013) |
| **BMPR1B** | rs1434536  C>T | miR-125b | OR | 1.72/TT | Increase | Breast cancer | (Saetrom et al, 2009) |
|  |  |  |  | 1.60 (1.01-2.53)/TT+CT |  | Prostate cancer | (Feng et al, 2012) |
|  |  |  |  | 0.68 (0.50–0.91)/TT+CT |  | Endometriosis | (Chang et al, 2013) |
|  | rs1970801  C>T |  |  | 0.47 (0.27–0.82)/TT |  |  |  |
| ***CASP3*** | rs1049253  T>C | miR-885-5p | OR | 1.80 (1.13–2.87)/CC+CT | Increase | Head and neck cancer | (Guan et al, 2013) |
| ***EFNA1*** | rs12904  G>A | miR-200c | OR | 0.65 (0.50–0.85)/AA | Increase | Gastric cancer | (Li et al, 2014) |
| **EGFR** | 774C>T | miR-214 | OR | 1.29 (1.05–1.58)/TT | Increase | Bladder cancer | (Chu et al, 2013) |
| **HOXB5** | rs9299  A>G | miR-7 | OR | 6.07 (2.99–12.31)/GG | Increase | Bladder cancer | (Luo et al, 2012) |
| ***IL-1a*** | rs3783553  0bp>4bp | miR-122 | OR | 1.5 (1.1-2.2)/InsIns | Increase | Oropharyngeal cancer | (Zhang et al, 2015) |
|  |  |  |  | 0.62 (0.49–0.78)/InsIns |  | Hepatocellular carcinoma | (Gao et al, 2009) |
| **IQGAP1** | rs1042538  A>T | miR-124 | OR | 0.78 (0.61-0.99)/TA | Increase | Breast cancer | (Zheng et al, 2011) |
| **KIT** | rs17084733  G>A | miR-122 | OR | 4.24 (1.25-14.65)/AA | Increase | Acral melanoma | (Godshalk et al, 2011) |
| ***KRAS*** | rs61764370  T>G | let-7 | OR | 3.05 (1.53–6.08)/TT+GT | Increase | Gastric cancer | (Li et al, 2013) |
|  |  |  |  | 2.3 (1.1-4.6)/TT+GT |  | NSCLC | (Chin et al, 2008) |
|  |  |  |  | 1.5 (0.9-2.5)/TT+GT |  | Pharyngeal cancer | (Christensen et al, 2009) |
| **MYCL1** | rs3134615  G>T | miR-1827 | OR | 2.08 (1.39–3.21)/TT | Increase | SCLC | (Xiong et al, 2011) |
| **NFκBIA** | rs696  A>G | miR-449a | OR | 1.38 (1.14–1.66)/GG | Increase | Colorectal  cancer | (Song et al, 2011) |
| **PDGFC** | rs1425486  G>A | miR-425 | OS  (HR) | 2.69 (1.67-4.33)/AA | Increase | Ovarian cancer | (Liang et al, 2010) |
| ***PIK3CA*** | rs141178472  C>T | miR-520a | OR | 1.716 (1.084–2.716)/TT | Increase | Colorectal  cancer | (Ding et al, 2015) |

**Table EV3: Mutations & SNPs in miRNA binding sites in 3’UTRs associated with cancer (cont.)**

| **Gene** | **Variant** | **miRNA** | **Endpoint** | **Value of Endpoint** (95% CI) **/ Genotype** | **Expres-sion** | **Cancer type** | **Reference** |
| --- | --- | --- | --- | --- | --- | --- | --- |
| ***RAD52*** | rs7963551  A>C | let-7 |  | 0.67 (0.51–0.89)/CC+CA | Increase | HBV-related  hepatocellular carcinoma | (Li et al, 2015) |
|  |  |  |  | 0.49 (0.37–0.65)/CC+CA |  | Glioma | (Lu et al, 2014) |
|  |  |  |  | 0.84 (0.75-0.95)/CC |  | Breast cancer | (Jiang et al, 2013) |
| **RAP1A** | rs6573  A>C | miR-196a | OR | 0.43 (0.21–0.91)/CC+AC | Increase | Esophageal squamous cell carcinoma | (Wang et al, 2012) |
| **REV3L** | rs465646  T>C | miR-25  miR-32 | OR | 0.71 (0.59–0.85)/CC+TC | Increase | Lung cancer | (Zhang et al, 2013) |
| **RYR3** | rs1044129  A>G | miR-367 | OR | 1.26 (1.03–1.54)/GG+AG | Increase | Breast cancer | (Zhang et al, 2011) |
| ***SGSM3*** | rs56228771  0bp>4bp | miR-151-5p | OR | 0.55 (0.42–0.73)/InsIns+InsDel | Increase | Hepatocellular carcinoma | (Wang et al, 2014) |
| ***TLR4*** | rs1057317  C>A | miR-34a | OR | 1.278 (1.053–1.552)/AA | Increase | Hepatocellular carcinoma | (Jiang et al, 2014) |
| **BRCA1** | rs799917  C>T | miR-638 | OR | 1.95/TT | Decrease | Breast cancer | (Nicoloso et al, 2010) |
| **BTRCP** | rs16405  9bp>0bp | miR-920 | OR | 0.44 (0.24-0.83)/DelDel+InsDel | Decrease | Hepatocellular carcinoma | (Chen et al, 2010) |
| ***CD80*** | rs1599795  A>T | miR-132-3p  miR-212-3p  miR-361-5p | OR | 1.48 (1.03–2.12)/TT+TA | Decrease | Gastric cancer | (Wu et al, 2014) |
| **CD133** | rs2240688  A>C | miR-135a/b | OR | 0.80 (0.72-0.90)/CC | Decrease | Lung cancer | (Cheng et al, 2013b) |
| **KRT81** | rs3660  G>C | miR-17 | OS | Median not reached vs. 7.0 years/CC | Decrease | Multiple myeloma | (de Larrea et al, 2012) |
| **MBL2** | rs10082466  T>C | miR-27a/b | OR | 3.17 (1.57–6.40)/CC+CT | Decrease | Colon cancer | (Zanetti et al, 2012) |
| ***MDM4*** | rs4245739  A>C | miR-191-5p  miR-887-3p | OR | 0.45 (0.35–0.59)/CC+CA | Decrease | Breast cancer | (Liu et al, 2013) |
|  |  |  |  | 0.54 (0.32–0.89)/CC+CA  0.47 (0.26–0.88)†/CC+CA |  |  | (Gao et al, 2015) |
|  |  |  |  | 0.54 (0.35-0.82)/CC  0.68 ( 0.45-0.99)†/CC |  | Esophageal squamous cell carcinoma | (Zhou et al, 2013) |
|  |  |  | OR f. TRD | 5.5 (1.5–20.5)/AA+AC |  | Ovarian cancer | (Wynendaele et al, 2010) |
| ***MTHFR*** | rs114673809  C>T | miR-214 | OR | 1.667 (1.044–2.660)/CC+CT | Decrease | Gastric cancer | (Chen et al, 2015) |
| **MTMR3** | rs12537  C>T | miR-181a | OR | 1.72 (1.36–2.16)/TT | Decrease | Gastric cancer | (Lin et al, 2012) |
| **NBS1** | rs2735383  G>C | miR-629 | OR | 1.40 (1.18-1.66)/CC | Decrease | Lung cancer | (Yang et al, 2012) |
| **NPM1** | 1146delT | miR-337-5p | OS (months) | 9 vs. 12 | Decrease | Acute myeloid leukemia | (Cheng et al, 2013a) |

**Table EV3: Mutations & SNPs in miRNA binding sites in 3’UTRs associated with cancer (cont.)**

| **Gene** | **Variant** | **miRNA** | **Endpoint** | **Value of Endpoint** (95% CI) **/ Genotype** | **Expres-sion** | **Cancer type** | **Reference** |
| --- | --- | --- | --- | --- | --- | --- | --- |
| **SET8** | rs16917496  T>C | miR-502 | OR | 1.66 (1.06-2.61)/CC | Decrease | Breast cancer | (Song et al, 2009) |
|  |  |  | OS (months) | 58.0 vs 41.0/CC |  | NSCLC | (Xu et al, 2013a) |
|  |  |  | OS (HR) | 0.175 (0.053-0.577)/CC |  | Hepatocellular carcinoma | (Guo et al, 2012) |
| ***TNFAIP2*** | rs8126  T>C | miR-184 | OR | 1.48 (1.06-2.05)/CC | Decrease | Head and neck cancer | (Liu et al, 2011) |
|  |  |  |  | 2.00 (1.09-3.64)/CC |  | Gastric cancer | (Xu et al, 2013b) |
| **XPO5** | rs11077  A>C | miR-4763-5p | OS | Median not reached vs. 7.2 years/CC+CA | Decrease | Multiple myeloma | (de Larrea et al, 2012) |

**References Table EV3**

Chang CY, Chen Y, Lai MT, Chang HW, Cheng J, Chan C, Chen CM, Lee SC, Lin YJ, Wan L, Tsai PW, Yang SH, Chung C, Sheu JJ, Tsai FJ (2013) BMPR1B up-regulation via a miRNA binding site variation defines endometriosis susceptibility and CA125 levels. *PLoS One* **8:** e80630

Chen Q, Qin R, Fang Y, Li H, Liu Y (2015) A Functional Variant at the miR-214 Binding Site in the Methylenetetrahydrofolatereductase Gene Alters Susceptibility to Gastric Cancer in a Chinese Han Population. *Cell Physiol Biochem* **36:** 622-630

Chen S, He Y, Ding J, Jiang Y, Jia S, Xia W, Zhao J, Lu M, Gu Z, Gao Y (2010) An insertion/deletion polymorphism in the 3' untranslated region of beta-transducin repeat-containing protein (betaTrCP) is associated with susceptibility for hepatocellular carcinoma in Chinese. *Biochem Biophys Res Commun* **391:** 552-556

Cheng CK, Kwan TK, Cheung CY, Ng K, Liang P, Cheng SH, Chan NP, Ip RK, Wong RS, Lee V, Li CK, Yip SF, Ng MH (2013a) A polymorphism in the 3'-untranslated region of the NPM1 gene causes illegitimate regulation by microRNA-337-5p and correlates with adverse outcome in acute myeloid leukemia. *Haematologica* **98:** 913-917

Cheng M, Yang L, Yang R, Yang X, Deng J, Yu B, Huang D, Zhang S, Wang H, Qiu F, Zhou Y, Lu J (2013b) A microRNA-135a/b binding polymorphism in CD133 confers decreased risk and favorable prognosis of lung cancer in Chinese by reducing CD133 expression. *Carcinogenesis* **34:** 2292-2299

Chin LJ, Ratner E, Leng S, Zhai R, Nallur S, Babar I, Muller RU, Straka E, Su L, Burki EA, Crowell RE, Patel R, Kulkarni T, Homer R, Zelterman D, Kidd KK, Zhu Y, Christiani DC, Belinsky SA, Slack FJ, Weidhaas JB (2008) A SNP in a let-7 microRNA complementary site in the KRAS 3' untranslated region increases non-small cell lung cancer risk. *Cancer Res* **68:** 8535-8540

Christensen BC, Moyer BJ, Avissar M, Ouellet LG, Plaza SL, McClean MD, Marsit CJ, Kelsey KT (2009) A let-7 microRNA-binding site polymorphism in the KRAS 3' UTR is associated with reduced survival in oral cancers. *Carcinogenesis* **30:** 1003-1007

Chu H, Wang M, Jin H, Lv Q, Wu D, Tong N, Ma L, Shi D, Zhong D, Fu G, Yuan L, Qin C, Yin C, Zhang Z (2013) EGFR 3'UTR 774T>C polymorphism contributes to bladder cancer risk. *Mutagenesis* **28:** 49-55

de Larrea CF, Navarro A, Tejero R, Tovar N, Diaz T, Cibeira MT, Rosinol L, Ferrer G, Rovira M, Rozman M, Monzo M, Blade J (2012) Impact of MiRSNPs on survival and progression in patients with multiple myeloma undergoing autologous stem cell transplantation. *Clin Cancer Res* **18:** 3697-3704

Ding L, Jiang Z, Chen Q, Qin R, Fang Y, Li H (2015) A functional variant at miR-520a binding site in PIK3CA alters susceptibility to colorectal cancer in a Chinese Han population. *Biomed Res Int* **2015:** 373252

Feng N, Xu B, Tao J, Li P, Cheng G, Min Z, Mi Y, Wang M, Tong N, Tang J, Zhang Z, Wu H, Zhang W, Wang Z, Hua L (2012) A miR-125b binding site polymorphism in bone morphogenetic protein membrane receptor type IB gene and prostate cancer risk in China. *Mol Biol Rep* **39:** 369-373

Gao F, Xiong X, Pan W, Yang X, Zhou C, Yuan Q, Zhou L, Yang M (2015) A Regulatory MDM4 Genetic Variant Locating in the Binding Sequence of Multiple MicroRNAs Contributes to Susceptibility of Small Cell Lung Cancer. *PLoS One* **10:** e0135647

Gao Y, He Y, Ding J, Wu K, Hu B, Liu Y, Wu Y, Guo B, Shen Y, Landi D, Landi S, Zhou Y, Liu H (2009) An insertion/deletion polymorphism at miRNA-122-binding site in the interleukin-1alpha 3' untranslated region confers risk for hepatocellular carcinoma. *Carcinogenesis* **30:** 2064-2069

Gartner JJ, Parker SC, Prickett TD, Dutton-Regester K, Stitzel ML, Lin JC, Davis S, Simhadri VL, Jha S, Katagiri N, Gotea V, Teer JK, Wei X, Morken MA, Bhanot UK, Chen G, Elnitski LL, Davies MA, Gershenwald JE, Carter H, Karchin R, Robinson W, Robinson S, Rosenberg SA, Collins FS, Parmigiani G, Komar AA, Kimchi-Sarfaty C, Hayward NK, Margulies EH, Samuels Y (2013) Whole-genome sequencing identifies a recurrent functional synonymous mutation in melanoma. *Proc Natl Acad Sci U S A* **110:** 13481-13486

Godshalk SE, Paranjape T, Nallur S, Speed W, Chan E, Molinaro AM, Bacchiocchi A, Hoyt K, Tworkoski K, Stern DF, Sznol M, Ariyan S, Lazova R, Halaban R, Kidd KK, Weidhaas JB, Slack FJ (2011) A Variant in a MicroRNA complementary site in the 3' UTR of the KIT oncogene increases risk of acral melanoma. *Oncogene* **30:** 1542-1550

Guan X, Liu Z, Liu H, Yu H, Wang LE, Sturgis EM, Li G, Wei Q (2013) A functional variant at the miR-885-5p binding site of CASP3 confers risk of both index and second primary malignancies in patients with head and neck cancer. *Faseb j* **27:** 1404-1412

Guo Z, Wu C, Wang X, Wang C, Zhang R, Shan B (2012) A polymorphism at the miR-502 binding site in the 3'-untranslated region of the histone methyltransferase SET8 is associated with hepatocellular carcinoma outcome. *Int J Cancer* **131:** 1318-1322

Jiang Y, Qin Z, Hu Z, Guan X, Wang Y, He Y, Xue J, Liu X, Chen J, Dai J, Jin G, Ma H, Wang S, Shen H (2013) Genetic variation in a hsa-let-7 binding site in RAD52 is associated with breast cancer susceptibility. *Carcinogenesis* **34:** 689-693

Jiang ZC, Tang XM, Zhao YR, Zheng L (2014) A functional variant at miR-34a binding site in toll-like receptor 4 gene alters susceptibility to hepatocellular carcinoma in a Chinese Han population. *Tumour Biol* **35:** 12345-12352

Li Y, Nie Y, Cao J, Tu S, Lin Y, Du Y, Li Y (2014) G-A variant in miR-200c binding site of EFNA1 alters susceptibility to gastric cancer. *Mol Carcinog* **53:** 219-229

Li Z, Guo Y, Zhou L, Ge Y, Wei L, Li L, Zhou C, Wei J, Yuan Q, Li J, Yang M (2015) Association of a functional RAD52 genetic variant locating in a miRNA binding site with risk of HBV-related hepatocellular carcinoma. *Mol Carcinog* **54:** 853-858

Li ZH, Pan XM, Han BW, Guo XM, Zhang Z, Jia J, Gao LB (2013) A let-7 binding site polymorphism rs712 in the KRAS 3' UTR is associated with an increased risk of gastric cancer. *Tumour Biol* **34:** 3159-3163

Liang D, Meyer L, Chang DW, Lin J, Pu X, Ye Y, Gu J, Wu X, Lu K (2010) Genetic variants in MicroRNA biosynthesis pathways and binding sites modify ovarian cancer risk, survival, and treatment response. *Cancer Res* **70:** 9765-9776

Lin Y, Nie Y, Zhao J, Chen X, Ye M, Li Y, Du Y, Cao J, Shen B, Li Y (2012) Genetic polymorphism at miR-181a binding site contributes to gastric cancer susceptibility. *Carcinogenesis* **33:** 2377-2383

Liu J, Tang X, Li M, Lu C, Shi J, Zhou L, Yuan Q, Yang M (2013) Functional MDM4 rs4245739 genetic variant, alone and in combination with P53 Arg72Pro polymorphism, contributes to breast cancer susceptibility. *Breast Cancer Res Treat* **140:** 151-157

Liu Z, Wei S, Ma H, Zhao M, Myers JN, Weber RS, Sturgis EM, Wei Q (2011) A functional variant at the miR-184 binding site in TNFAIP2 and risk of squamous cell carcinoma of the head and neck. *Carcinogenesis* **32:** 1668-1674

Lu C, Chen YD, Han S, Wei J, Ge Y, Pan W, Jiang T, Qiu XG, Yang M (2014) A RAD52 genetic variant located in a miRNA binding site is associated with glioma risk in Han Chinese. *J Neurooncol* **120:** 11-17

Luo J, Cai Q, Wang W, Huang H, Zeng H, He W, Deng W, Yu H, Chan E, Ng CF, Huang J, Lin T (2012) A microRNA-7 binding site polymorphism in HOXB5 leads to differential gene expression in bladder cancer. *PLoS One* **7:** e40127

Nicoloso MS, Sun H, Spizzo R, Kim H, Wickramasinghe P, Shimizu M, Wojcik SE, Ferdin J, Kunej T, Xiao L, Manoukian S, Secreto G, Ravagnani F, Wang X, Radice P, Croce CM, Davuluri RV, Calin GA (2010) Single-nucleotide polymorphisms inside microRNA target sites influence tumor susceptibility. *Cancer Res* **70:** 2789-2798

Saetrom P, Biesinger J, Li SM, Smith D, Thomas LF, Majzoub K, Rivas GE, Alluin J, Rossi JJ, Krontiris TG, Weitzel J, Daly MB, Benson AB, Kirkwood JM, O'Dwyer PJ, Sutphen R, Stewart JA, Johnson D, Larson GP (2009) A risk variant in an miR-125b binding site in BMPR1B is associated with breast cancer pathogenesis. *Cancer Res* **69:** 7459-7465

Song F, Zheng H, Liu B, Wei S, Dai H, Zhang L, Calin GA, Hao X, Wei Q, Zhang W, Chen K (2009) An miR-502-binding site single-nucleotide polymorphism in the 3'-untranslated region of the SET8 gene is associated with early age of breast cancer onset. *Clin Cancer Res* **15:** 6292-6300

Song S, Chen D, Lu J, Liao J, Luo Y, Yang Z, Fu X, Fan X, Wei Y, Yang L, Wang L, Wang J (2011) NFkappaB1 and NFkappaBIA polymorphisms are associated with increased risk for sporadic colorectal cancer in a southern Chinese population. *PLoS One* **6:** e21726

Wang C, Zhao H, Zhao X, Wan J, Wang D, Bi W, Jiang X, Gao Y (2014) Association between an insertion/deletion polymorphism within 3'UTR of SGSM3 and risk of hepatocellular carcinoma. *Tumour Biol* **35:** 295-301

Wang K, Li J, Guo H, Xu X, Xiong G, Guan X, Liu B, Li J, Chen X, Yang K, Bai Y (2012) MiR-196a binding-site SNP regulates RAP1A expression contributing to esophageal squamous cell carcinoma risk and metastasis. *Carcinogenesis* **33:** 2147-2154

Wu R, Li F, Zhu J, Tang R, Qi Q, Zhou X, Li R, Wang W, Hua D, Chen W (2014) A functional variant at miR-132-3p, miR-212-3p, and miR-361-5p binding site in CD80 gene alters susceptibility to gastric cancer in a Chinese Han population. *Med Oncol* **31:** 60

Wynendaele J, Bohnke A, Leucci E, Nielsen SJ, Lambertz I, Hammer S, Sbrzesny N, Kubitza D, Wolf A, Gradhand E, Balschun K, Braicu I, Sehouli J, Darb-Esfahani S, Denkert C, Thomssen C, Hauptmann S, Lund A, Marine JC, Bartel F (2010) An illegitimate microRNA target site within the 3' UTR of MDM4 affects ovarian cancer progression and chemosensitivity. *Cancer Res* **70:** 9641-9649

Xiong F, Wu C, Chang J, Yu D, Xu B, Yuan P, Zhai K, Xu J, Tan W, Lin D (2011) Genetic variation in an miRNA-1827 binding site in MYCL1 alters susceptibility to small-cell lung cancer. *Cancer Res* **71:** 5175-5181

Xu J, Yin Z, Gao W, Liu L, Yin Y, Liu P, Shu Y (2013a) Genetic variation in a microRNA-502 minding site in SET8 gene confers clinical outcome of non-small cell lung cancer in a Chinese population. *PLoS One* **8:** e77024

Xu Y, Ma H, Yu H, Liu Z, Wang LE, Tan D, Muddasani R, Lu V, Ajani JA, Wang Y, Wei Q (2013b) The miR-184 binding-site rs8126 T>C polymorphism in TNFAIP2 is associated with risk of gastric cancer. *PLoS One* **8:** e64973

Yang L, Li Y, Cheng M, Huang D, Zheng J, Liu B, Ling X, Li Q, Zhang X, Ji W, Zhou Y, Lu J (2012) A functional polymorphism at microRNA-629-binding site in the 3'-untranslated region of NBS1 gene confers an increased risk of lung cancer in Southern and Eastern Chinese population. *Carcinogenesis* **33:** 338-347

Zanetti KA, Haznadar M, Welsh JA, Robles AI, Ryan BM, McClary AC, Bowman ED, Goodman JE, Bernig T, Chanock SJ, Harris CC (2012) 3'-UTR and functional secretor haplotypes in mannose-binding lectin 2 are associated with increased colon cancer risk in African Americans. *Cancer Res* **72:** 1467-1477

Zhang L, Liu Y, Song F, Zheng H, Hu L, Lu H, Liu P, Hao X, Zhang W, Chen K (2011) Functional SNP in the microRNA-367 binding site in the 3'UTR of the calcium channel ryanodine receptor gene 3 (RYR3) affects breast cancer risk and calcification. *Proc Natl Acad Sci U S A* **108:** 13653-13658

Zhang S, Chen H, Zhao X, Cao J, Tong J, Lu J, Wu W, Shen H, Wei Q, Lu D (2013) REV3L 3'UTR 460 T>C polymorphism in microRNA target sites contributes to lung cancer susceptibility. *Oncogene* **32:** 242-250

Zhang Y, Sturgis EM, Sun Y, Sun C, Wei Q, Huang Z, Li G (2015) A functional variant at miRNA-122 binding site in IL-1alpha 3' UTR predicts risk and HPV-positive tumours of oropharyngeal cancer. *Eur J Cancer* **51:** 1415-1423

Zheng H, Song F, Zhang L, Yang D, Ji P, Wang Y, Almeida M, Calin GA, Hao X, Wei Q, Zhang W, Chen K (2011) Genetic variants at the miR-124 binding site on the cytoskeleton-organizing IQGAP1 gene confer differential predisposition to breast cancer. *Int J Oncol* **38:** 1153-1161

Zhou L, Zhang X, Li Z, Zhou C, Li M, Tang X, Lu C, Li H, Yuan Q, Yang M (2013) Association of a genetic variation in a miR-191 binding site in MDM4 with risk of esophageal squamous cell carcinoma. *PLoS One* **8:** e64331

Zu Y, Ban J, Xia Z, Wang J, Cai Y, Ping W, Sun W (2013) Genetic variation in a miR-335 binding site in BIRC5 alters susceptibility to lung cancer in Chinese Han populations. *Biochem Biophys Res Commun* **430:** 529-534
